# Bodily-sensory modalities are linguistic modalities: supporting participation of people with congenital deafblindness in cultural language practice through insights from the protactile movement and material engagement theory

**DOI:** 10.3389/fpsyg.2026.1709038

**Published:** 2026-07-08

**Authors:** Kirsten Costain, Steve Rose

**Affiliations:** 1 Department of Combined Sensory Loss and Deafblindness – Congenital, Division 4: Sign Language and Deafblindness, Norwegian National Special Education Advisory Service - Statped, Oslo, Norway; 2 National Resource Centre for Deafblindness, Tromsø, Norway; 3 Centre of Excellence Deafblind, Able Australia, Melbourne, VIC, Australia; 4 Vision Australia, Melbourne, VIC, Australia

**Keywords:** congenital deafblindness, enactive embodiment, languaging, protactile language, tactile sign language

## Abstract

**Introduction:**

This article suggests ways in which a cultural sign language frame for use by and with people with congenital deafblindness might be supported. It proposes including an approach to tactile signing informed by insights from the protactile movement, which grew out of attempts to rework the visual nature of American Sign Language, thereby creating a new tactile language. Protactile language represents a profound commitment to a co-constructive, participatory and enactive form of tactile languaging that goes far beyond improved access to cultural linguistic signs and signing in the tactile modality, extending instead into tactile collaborative meaning-making at a deeper cognitive (conceptual-exploratory) level. Within an enactive embodiment perspective, both cognition and language are activities pursued by embodied subjects in real time, rather than abstract, propositional processes. Language of any kind is always with and from the body, and the sensory modalities of its use must be accessible to speakers and listeners.

**Methods:**

This article uses perspectives from Material Engagement Theory. We describe two examples from our own (non-protactile) practice and examine these through the lens of protactile language.

**Results:**

We suggest that attending to issues of tactile salience and reciprocity that are grammatically enacted in protactile language is valuable for co-constructive signing with people with congenital deafblindness.

**Discussion:**

Considering the principles of protactile language can enable conversation partners to move from a signal-based, information-relaying frame toward a reciprocal, dialogical communicative frame.

## 1 Introduction: congenital deafblindness (cdb) and barriers to cultural language

We are consultants working in the field of deafblindness with people with cdb with an interest in the study of meaning construction and language acquisition among this group of people. This article focuses on language practice in communication with people with cdb (People w/cdb) using sign language elements (visual manual) in the tactile modality (meaning mode of articulation of signs), and the potential contributions to this practice of principles of co-constructive meaning construction from *protactile* language (granda and Nuccio, 2018; Edwards and Brentari, 2020). Readers are referred to more detailed descriptions of sign language and to original sources describing protactile for an introduction to the history and philosophical movement of Protactile, of which the language is but one part. Our aim is to invite the reader practitioner to reflect on how protactile differs from tactilized sign language, and how we might think about these differences in a way that is actionable with People w/cdb. We suggest that protactile principles can offer conceptual clarity and depth to practices of co-signing and tactile communication. For both the practitioner and non-practitioner alike, the topic of language in the tactile modality itself highlights language in every modality as an embodied phenomenon, as something we do, and something we do together as shared meaning creation.

Visual and/or auditory function is usually present (“residual” sensory function) but severely limited in cdb. In rare cases, both senses are completely absent from birth. While remaining an important channel of experience and communication, one residual sense cannot compensate for the absent or severely reduced sense. Residual senses cannot be used dynamically in the taken-for-granted flow of perception experienced when function is intact (Nordic Welfare Centre, 2024). Because of the complexity of reduced sensory function in each individual with cdb and the heterogeneity that characterizes this group, each person w/cdb will have a highly individual experience of any residual distal sensory function. This negatively impacts their access to perception of others’ linguistic expressiveness, and thus to the phonology of cultural language, severely limiting opportunities to learn its articulation (Dammeyer and Ask Larsen, 2016; Larsen and Dammeyer, 2021).

Throughout this article, we will use the term cultural language and associated terms linguistic, linguistic symbolic system, and grammatical system to refer to languages that are formally recognized as consensual cultural languages in use by specific communities. These include national languages (Norwegian Sign Language, Urdu, and French) and have a grammatical structure and rules for usage, including those applying to articulation. That being said, a use perspective of language (Dufva, 2011; Evans, 2014; Tomasello, 1992), along with that of language as an embodied phenomenon that is ultimately an activity rather than a “thing” (Dufva, 2011; Gallagher, 2023), are our primary perspectives. Cultural languages exist through continued consensus about their use, but they are necessarily always changing and evolving precisely because they are used. As anyone who has ever tried to do conversation analysis will attest, speakers (and here we will use this term also with reference to sign languages) use cultural language in a creative, or generative manner (Evans, 2014; Evans et al., 2018; Du Bois and Giora, 2014). They bend or flout grammatical rules, play with the dictionary meanings of words, develop slang terms and new dialects, and use grammatical structures in a dynamic, often subversive and generative way to make points, develop ideas, and position themselves in relation to listeners, among many other achievements. We recognize that, although language is far more than culturally consensual grammaticalized and rule-defined systems, access to these systems is important in determining whether and to what extent communicators have access to those around them in the mainstream seeing and hearing culture. This access is severely limited for People w/cdb.

Increasingly, People w/cdb have multiple disabilities that place restrictions beyond deafblindness on their independent movement in the environment (Dammeyer, 2011). This includes the ability to move and/or manipulate extremities and limbs, including the fingers and hands, problems of muscular hypotonia, and barriers caused by complex and chronic medical conditions. Further complications include tactile defensiveness through heightened sensitivity to touch, including rejection of hand-to-hand contact with communication partners and objects (Costain, 2020; Larsen and Dammeyer, 2021).

Achievement of a strong enough commitment to bodily-tactile communication relevant for People w/cdb requires exchanging a visual logic with a proprioceptive-kinesthetic perceptual logic. A protactile logic highlights the salience of grounded co-construction of signs in contact space (the body of the “listener”) over form production (the completed sign or handshape focus of visual sign language) in air space (granda and Nuccio, 2018; Edwards and Brentari, 2020). We suggest that language use with and by People w/cdb is likely to be supported only through commitment to using this modality consistently in communication and interaction, and that incorporation of the principles of protactile can expand approaches to achieving this. Our practice examples are from previous case studies and have been reassessed following our recent interest in protactile theory. Our overarching methodological perspectives are “4-E” phenomenological accounts of embodiment (Gallagher, 2023) and the embodied cultural anthropological perspective of Material Engagement Theory—MET (Malafouris, 2008). We wish to suggest, through our brief account of MET, a further way of understanding the added value that principles of co-constructive tactile meaning construction in Protactile language may bring to communication between People w/cdb and their partners. We connect several other concepts from the phenomenology of language and cognition with relevance to the central arguments of these main perspectives.

## 2 People with congenital deafblindness: people w/cdb

There are differences between the perspectives of DeafBlind people who have both a cultural-linguistic and a visual and/or auditory history who transfer to tactile language from visual sign language, and those with cdb who have never had access to linguistic input sufficient for the acquisition of a cultural language. Those acquiring deafblindness after establishing language are also referred to as postlingual-deafblind, and those with cdb as prelingual-deafblind using language onset as the distinction between groups (Dammeyer, 2012).

We have chosen to denote the group of people with congenital deafblindness as People w/cdb. Unlike DeafBlind people, among whom are the initial developers of Protactile language (granda and Nuccio, 2018), People w/cdb have no sociopolitical identity, and it would be disingenuous to imply that they do. Defining the visual and auditory status of children who are encompassed by this definition is not irrelevant or discriminatory, but rather the opposite. It constructs them as members of an identifiable group so that they can be considered and brought into the protactile conversation in the first place. Put simply, DeafBlind people are not People w/cdb, and children w/cdb are not very young DeafBlind people.

### 2.1 Definitions of cdb

Research in the field of deafblindness generally and in terms of cdb specifically remains hindered by contradictory definitions, assessment challenges, and the heterogeneity of the populations of congenital and acquired deafblindness (Larsen and Dammeyer, 2021). The definition of congenital deafblindness that we employ in this article and practice is as follows: combined visual and auditory loss or reduced function present at birth or appearing in the first year of such character and severity as to prevent sufficient access to linguistic culture for language development to begin (Ask Larsen and Damen, 2014; Dammeyer, 2008). In using this definition, we ally ourselves most with the Nordic Definition of Deafblindness (ability/functioning) (Nordic Welfare Centre, 2024). This states that the total functional consequences of the dual sensory disability are considered for four areas of functioning: communication, access to information, orientation and mobility, and participation. This definition distinguishes congenital deafblindness from acquired deafblindness based on developmental age (Dammeyer, 2011; Larsen and Dammeyer, 2021). While causes of deafblindness in both populations are congenital, dual sensory disability in the acquired group becomes deafblindness over a longer span of time, after language acquisition. DeafBlind people have a visual and/or auditory past and cultural linguistic history such that protactile is a second, and sometimes third, language for them (Gagne et al., 2023).

Among People w/cdb, multiple disability resulting from complex etiologies is the norm (Dammeyer, 2011; Rødbroe and Janssen, 2006). Very few have complete deafblindness, so residual visual and/or auditory function is common. Despite this, reduced sensory function is highly fragmentary and idiosyncratic, and often complicated further by vestibular, tactual, and other disability. Many have intact or relatively mild peripheral sensory disability of one or both distal senses, accompanied by significant central processing disability.

There are no standardized instruments for assessing children with deafblindness, and the few surveys of cdb that have been conducted have not provided evidence of correlation between the degree of sensory impairment and cognitive or communicative delay (Larsen and Dammeyer, 2021). Tosolini et al. (2025) performed a scoping review of 13 studies of children w/cdb that used definitions of cognition compatible with those of Piagetian coping skills, such as goal-directed behaviors and object permanence. They found that engaging with objects independently rarely occurs and that children w/cdb have fewer, more restricted, and qualitatively different opportunities to act upon their environments than do hearing and seeing children. People w/cdb are dependent on 24-h care and the presence of a support partner. Communication is usually multimodal, and the tactile sense remains the most intact communicative channel for the Person w/cdb (Dammeyer, 2015). Tactile function is often complicated by hyper- or over-sensitivity to touch, a situation that can rapidly develop into tactual impairment and further isolation through deprivation (Costain, 2020). Nevertheless, the bodily-tactile sense (tactile = touch, proprioceptive = bodily positioning in space, kinesthetic = action-movement senses) is foundational for People w/cdb (Dammeyer and Nielsen, 2013).

### 2.2 Research on communication and cdb

Qualitative case studies using video analysis have formed the bulk of work in language development and communication of and with People w/cdb. These have tended to focus more globally on communication, intersubjectivity, and prelinguistic expressivity (i.e., Bruce, 2005; Rødbroe and Janssen, 2006; Dammeyer and Nielsen, 2013; Nafstad and Rødbroe, 2015). Among sign linguistic studies is Forsgren et al. (2018), who identified a new category of authentic sign based on heightened tactile sensitivity. There is renewed interest in the notion of tactile language, and conversation analysis applied to filmed interactions between People w/cdb and their partners (Creutz et al., 2019; Dammeyer et al., 2015).

Two recent studies will serve to characterize current interests in this area. Peltokorpi et al. (2024), in a single case replication study, looked at play interactions between mothers and their young children with visual impairment and additional disabilities. They found that following coaching, mothers learned ways of using touch and movement (the “bodily-tactile modality”) to make their interactions with the children more accessible and helped them identify children’s bodily expressions and respond to these using touch. This involved modeling different communicative functions of touch for mothers: noticing a child’s expressive movement through touch; anticipation (mother touching a child’s hands before grasping them); and using bodily-tactile imitation to enhance turn-taking. Co-active signing (adult guiding the child’s hands) and body signs (signs made directly on the child’s body) were introduced as sign-lexical components. Mothers’ increased use of the bodily-tactile modality during the intervention reflected results of prior research (Peltokorpi et al., 2023, 2020). Noticing children’s actions through touch seemed to make parents’ responsiveness more accessible to their children (Peltokorpi et al., 2024).

Warnicke et al. (2024) used video analysis and multimodal conversation analysis (Mondada, 2014) to examine interactional practices aimed at orientation between an adult w/cdb and his support partners. Multimodal resources in the study included actions, movement, gaze, body posture, facial expressions, gestures, and intentional communicative use of material contexts, such as objects. Reciprocity in neutral understanding of activities, time, space, and actors was achieved through repetition of signs in a second turn and repetition of a sign placed first in a question-answer adjacency pair, as well as a visual schedule (Warnicke et al., 2024).

Two recent studies used protactile principles with children with cdb. Lu et al. (2024) explored the use of touch and protactile indexical signing with two DeafBlind toddlers in their homes. The researcher aimed to establish intersubjective attunement to meaningfully connect signs, her intentions and her actions. Adopting the peripheral structure of rooms as starting points for orientation created contextualized sensory access to a meaningful environment that included paths to destinations and activities in which partners could operate in an intersubjectively attuned manner on and with objects.

Establishing a shared “relational tactile ground” is necessary to perceive emotion and communication responses, allowing partners to distinguish between purposeful action and self-regulatory behavior (Lu et al., 2024; granda and Nuccio, 2018). Gagne et al. (2023) found in a case study with a child with cdb that observing contact space in tactile interactions created co-presence while engaged in object exploration (adult’s hand + child’s hand + object), protactile descriptions (hand to arm), and labeling after mutual contact with an object. In protactile, intersubjective attunement, shared tactile exploration and articulatory processes are facets of the same activity.

### 2.3 People w/cdb and lack of cultural linguistic language development

Access and movement restrictions in cdb represent barriers to cultural language exposure and development for which People w/cdb have received far too little support, effectively creating a situation of cultural language deprivation for the majority (Dammeyer and Ask Larsen, 2016). The obvious perceptual barriers to visuospatial development, coupled with few, insufficient, and/or erratic opportunities for participation in structured activities, reduce access to the central “plot” schematic of the human act (source-path-goal; Bruner, 1986; Johnson, 2010). This narrative structure forms the basis of human conceptual development (McGowan and Delafield-Butt, 2022). These barriers are significant factors in accounting for the lack of cultural language use by and with People w/cdb. Without activities and situations in which to use it, exposure to cultural language in any consistent manner is absent or limited. Cultural signs in the tactile modality tend to be used in practice (in our experience) as one, often least-favored element in a mix drawn from both technological and manual-tactual forms of Augmentative and Alternative Communication (AAC).^1^ Exposure to manual signing is sometimes through restricted manual communication systems (sign interventions) that have their own vocabularies, such as Makaton™ or Key Word Signing (Grove and Launonen, 2019), which are distinct from Sign Languages.

Another potential reason for the lack of cultural language development is suggested by case studies of gesture use by a man with complex brain injury (Gallagher, 2005; McNeill, 2005). Two forms of gestural hand movement were identified: topokinetic (movement aspects to do with precision regarding spatial location and accuracy of movement to targeted external points) and morphokinetic (aspects having to do with shape or form regardless of spatial location) (Gallagher, 2005). While gesturing relies mostly on morphokinetic accuracy (*Ibid.*), cultural sign language can be seen to rely heavily on topokinetic targeted movement. This is because sign language requires good hand-eye coordination as well as fine motor control and manual skill (also in the tactile modality when the speaker is DeafBlind). As visual languages, sign languages are based on a visual understanding of space and spatial relationships (Emmorey, 2001). Signs are distinct from gestures, and all language users employ gestures in addition to and during linguistic communication (Emmorey, 2001; Jagoe and Wharton, 2021). “Home signs” of young deaf children of non-Deaf parents are easily distinguished as “purely gestural” when compared with the precision of movement and handshape of cultural language signs (Grove and Launonen, 2019). The directed movements of cultural sign languages require a significant level of precision that most People w/cdb have little opportunity to perceive, also when signs are made tactile.

Morphokinetic, form-related, and less accurate movements are seen in all human gesturing and appear to be largely independent of the systems controlling instrumental movement (Gallagher, 2005). They are related to the meaning and storytelling aspects of gesture accompanying speech, and this connects to the primacy of movement and of narrative for the development of human cognition (Delafield-Butt and Trevarthen, 2015). Gesture though, like cultural language, is a particular form of expressive movement and shares its origin with all other movement-borne “forms of vitality” of the human being (Stern, 2010; movement and its “four daughters,” force, time, space, and directionality) and in the “implicit motive pulse,” with which we are born, the psychobiological foundation for the shaping of human movement processes, from locomotion to learning, in narrative form (Malloch and Trevarthen, 2009). Like all directed movement, expressive movement in signing is movement through space and involves a linear path structure of start-middle-end or source-path-goal (Johnson, 1987). Concepts of linearity and sequentiality require experiences of the path variety: cause and effect, stop and start, moving from ‘here’ to get to ‘there’, figure and ground (*Ibid.*). For People w/cdb, these experiences are likely to be far fewer than for people with better distal access, and often highly fragmented (Dammeyer, 2011; Larsen and Dammeyer, 2021).

### 2.4 Radical embodiment: language and cognition from the body

The linguistic frame for this article is that of sign language. All languages rely on bodily movement: vocal and sign languages are movement systems at their base. Cultural languages are symbolic, formal linguistic systems with vocabularies, phonology, grammar, rule-based usage, and dialects. As well, in active use by speakers/signers, languages are systems of articulative movement. Signers move their hands, fingers, wrists, arms, faces, heads, and bodies. Vocal speakers articulate through shaping breath streams with their tongue, mouth, and jaw muscles, and by moving air over their vocal cords. Speech therapist Gilman (2014) goes further in describing how the voice involves the whole body in a complex coordination of respiration, phonation, and resonance systems, including skeletal, muscular, and neurological systems. Language is ever only real, as Wittgenstein (1976) remarked, when in active use, and this use is always in terms of bodily movement.

This focus on movement has also informed 4E theories of cognition (embodied, enactive, embedded, and extended) and of psychological development in terms of embodied processes that include, but are not restricted to, the brain (Gallagher, 2023). These theories range from “weak” to “strong” roles for the extra-neural body in thinking and conceptual knowledge creation and use (Alessandroni et al., 2024; Gallagher, 2023). At the radical, enactive-participatory end of the spectrum, cognition, like language, is movement-based, becoming action in terms of intentional and directed movement of the organism serving both non-propositional as well as propositional activities (Gallagher, 2011). Intention at the level of pre-reflective awareness is implied by movement and is the distinguishing feature of being alive (Gallagher, 2005; Sheets-Johnstone, 1998).

Werner and Kaplan (1963) suggested that the connection of early dynamic responses, such as head- and gaze turning to auditory stimuli, is the basis of emerging symbolic understanding in what they called physiognomic perception, or perceiving the actions of the mind in outward, physical action (Stern, 2010). There is no need for the child to develop a “theory” of mind for intersubjectivity to begin, in other words, because the following of the movements of others begins from birth in the earliest face-to-face encounters with caregivers (De Jaegher et al., 2010; Gallagher, 2005). Parents and infants learn to read each other’s “body language,” and cultural linguistic language systems share this same root and form; they, too, are languages from the body, the only kind there are.

#### 2.4.1 Language as languaging

In “use” theories of language (Dufva, 2011; Evans, 2014; Tomasello, 1992), then, language is really languaging, in that it exists as such through the doing of it (García and Wei, 2014). Cultural languages involve special kinds of movements distinct to the bodily mode of articulation (vocal, manual) of each linguistic system. Language as a broad phenomenon beyond cultural languages can also be thought of as expressive movement in the sense of what Mead (1934) called intentional gesture, directed at calling forth a response from the other. Whether these symbols become cultural-linguistic or not has to do with consensus in the practice of their use over time (and the rule-based nature of this practice—grammar; Evans, 2014). However, cultural or not, their origins will lie in embodied experience. Although the symbols of established cultural languages may seem arbitrary, they too have their origins in the physical world and in real-life actions. Languaging is an activity that includes the use of linguistic symbols, whether as manual signs or vocal words. If we extend languaging to include symbolic communication also outside of cultural-linguistic systems, we can view the authentic and/or non-cultural-linguistic bodily expressions of People w/cdb as participation in this activity.

Sign languages are visual-manual languages (Woll and Sutton-Spence, 2023) and, as such, they afford the presentation of parallel streams of information and context (Emmorey, 2001). Signing is part of a discursive spatial context that includes non-manual features such as facial expressions and the reactions of the listener that are simultaneously perceivable (Jepsen et al., 2015). In tactile form, this parallel feature disappears (Willoughby et al., 2018, p.25).

Tactile exploration processes are sequential, as one piece of information must be explored before moving on to the next aspect (e.g., temperature, texture, shape, weight, or size; Lederman and Klatzky, 1987). Movement, one of the linguistic features of a sign, is in terms of path (trajectory, also showing orientation, another sign linguistic quality) or finger−/hand movements (Emmorey, 2001). The linearity that makes path movement understandable, as such, is not a schematic that is easily accessible to People w/cdb and an area in which many People w/cdb lack consistent support.

## 3 Cultural signing by people w/cdb

### 3.1 Emblematic use of cultural signs by people w/cdb

Despite the much-cited problem of “the struggle to symbolism” (the awareness of abstraction from the concrete required for symbolic understanding) by People w/cdb (Bruce, 2005), our experience and that of other partners is that they frequently use their few cultural and/or negotiated authentic signs in an evocative, rather than directly referential way (for example, Costain et al., 2019). Jagoe and Wharton (2021) point out the lack of research into the potential communicative roles of gesture in non-propositional information provision, and indeterminate or vague communication, as well as the pervasiveness of the narrow decoding perspective of utterance interpretation in the field of linguistic pragmatics. They adopt Sperber and Wilson’s (1986) perspective on Grice's (1957) relevance theory model of communication. In Grice’s theory, communication is primarily the expression and recognition of a speaker’s intentions in the performance of an observable stimulus, instead of the sender–receiver model of communication in which a communicator encodes a thought into a signal that is then retrieved by the hearer after successful decoding of the “sent” signal. Sperber and Wilson take Grice’s emphasis on the speaker’s intention as one of specific modification of a hearer’s thoughts and expand this to characterize speaker intention as that of modifying a hearer’s cognitive environment (Sperber and Wilson, 1986). Grice distinguishes between “non-natural meaning” (communicated through a symbolic code, like a cultural language) and “showing” (for example, by pointing to a clock when asked the time). While showing is defined as having a non-symbolic specificity and therefore highly determinate, some acts of showing can be equally indeterminate. If, when hearing the time or being shown the clock, the questioner puts their hand to their forehead and sighs, this act of showing is not about a specific topic, but rather a suggestion of feelings and thoughts, and the intention is to convey an impression, rather than a particular meaning about “any one thing” (Jagoe and Wharton, 2021, p.25).

People w/cdb often use single elements from a limited sign repertoire in a similar suggestive and indeterminate way (Schjøll Brede, 2019), although their expressiveness is, in our experience, often interpreted by the support environment mostly in terms of highly determined instrumental demands, wishes, or needs.

In his review of various historical classifications of gesture (as an accompaniment to speech), Kendon (2004) describes a continuum of symbolism ranging from purely gestural movement to what he calls linguistic gesture:

Gesticulation→language-like gestures→pantomimes→emblems→sign languages.

He defines emblems (such as ‘the finger’ or the ‘OK’ sign) as entirely culture-dependent and highly “fixed” (in terms of meaning) symbolic gestures nevertheless used to convey a wide range of both positive and negative meanings. People, w/cdb’s use of cultural signs can be said to be similarly emblematic-evocative rather than determinate-referential. The sign for COFFEE can be a way of bringing in a whole scenario—one involving cups of coffee but also specific people, settings, allied activities within the setting or scenario, emotions, memories, and times of day or week. The intention might be a simple or a more complex request (for a cup of coffee, or and sometimes as well, a suggestion to revisit the emotions one had that other time in the coffee-drinking scenario); it might be a way of introducing an abstract theme, such as feelings of loneliness or boredom; it might be a way of expressing affection for the people implicated in the scenario, or a question about why they aren’t present when expected.

### 3.2 Expressiveness: what is ‘ready to hand’ gets used

People w/cdb are pragmatic in that they will use several modes and forms of expression and do so economically—what is “ready-to-hand” gets used (Heidegger, 1968). They often provide ample evidence of understanding the formal signs used in contexts that are meaningful to them and their partners, yet fail to produce much culturally signed expressive communication on their own initiative. Fluency of one’s own authentic expressions/expressiveness may be preferable to struggling to produce conventional signs in “air space” (granda and Nuccio, 2018), as expressiveness is language-as-way-of-being rather than a system or thing (Trevarthen and Aitken, 2001). Familiar concepts such as readability relate to the system/object view of language, whereas interpretation of expressiveness requires intersubjective following and accompanying, coordination, and engagement rather than decoding an encoded message (De Jaegher et al., 2010). A more fitting term reflecting this latter perspective and following protactile principles is “salience” (Edwards and Brentari, 2020; granda and Nuccio, 2018): when the perceivable specificity of an expression is high, interlocutors will be better able to follow each other into a process of sharing meaning.

## 4 Protactile language

### 4.1 Sign languages and protactile language

Sign languages are distinct cultural languages. The lexical features of the sign are handshape, movement (path and finger movement), location, orientation, and non-manual behaviors, such as facial expressions (Stokoe, 1960; Emmorey, 2001; Willoughby et al., 2018). Signs are made in sign space in front of the body of the signer. The two hands of the signer or “speaker” (the terms speaker and listener reflect current usage in accounts of protactile language; Gagne et al., 2023) are called articulators, with the dominant hand (H1) the most active articulator (movement, main handshape) and the non-dominant hand (H2) serving as the location part of the sign. Classifiers are a fixed set of handshapes adopted to simultaneously represent objects or other discrete aspects of the environment, spatial locations, and movement (Supalla, 1986; Woll and Sutton-Spence, 2023), e.g., a single person may be represented by a single upright finger, and a group represented by multiple fingers with direction of movement shown simultaneously using the classifier handshape. The person (index finger) or group (five-hand, fingers upright, palm forwards) can move forwards, backward, toward, or away. In the four-handed dyad of tactilized sign language, the speaker uses both “speaking-hands” and the listener places both their hands on these as “listening-hands” (Deuce and Rose, 2019; Willoughby et al., 2018).

Protactile language uses tactile modalities exclusively and is situated within an environment organized along lines of tactility that requires a tactile deictic system (Edwards and Brentari, 2021, 2020). It evolved as part of the protactile movement in the early 2010s in Seattle among a group of DeafBlind users of American Sign Language (ASL) who were concerned with their increased dependence on sighted interpreters, and found that tactilized ASL lacked the vitality and relevance it once had when they too had been sighted (Edwards and Brentari, 2021; granda and Nuccio, 2018). granda and Nuccio (2018) outline seven protactile principles that distinguish protactile language from tactualized-ASL (see Table 1). The social and political protactile movement of which protactile language is a part was built on the notion that vision and hearing are not necessary, in that all human activity can be redirected through tactile channels (Edwards, 2018, 2024). In recent years, there has been a shift away from tactile access towards discovering the maximal potential of tactile channels for all arenas of life (Clark and Nuccio, 2020; Edwards and Brentari, 2021; granda and Nuccio, 2018).

**Table 1:** Summary of protactile language principles (derived from granda and Nuccio, 2018, original text, with clarifications).

Principle/sub principle	Description
1. Contact space	Contact space is the body of the addressee. Any time space is used, make sure it is contact space, not ‘air space’ (p. 4).
1a. Reference markers	A point in contact space should be created when talking about people or things, which should subsequently be referred to consistently (p. 4).
1b. Role shift	In protactile, narrative positions between people in a story must be expressed in contact space (p. 4).
1c. Point to point	The first and last thing must be anchored in contact space when spatial relationships between two or more things are expressed (p. 5).
1d. Emphasis and emotion	Emphasis and emotion often expressed on the face should also be communicated in contact space. One option uses conventionalized mouth gestures or other facial expressions to create tactile representations. The second option adopts purely tactual expressions (reflecting characteristics of emotion in the tactile experience). Either approach is acceptable within contact space, not air space (p. 5–6).
2. Reciprocity	Always communicate reciprocally through touch regardless of how much you see (p. 7).
3. Protactile perspective	Internalize a protactile perspective by (i) working together to co-create signs that are easy to feel, and (ii) describing things in ways that reflect protactile experience (p. 8).
3a. Classifiers (in visual sign languages, signs that use handshapes associated with specific classes of things are described as ‘classifiers’)	Classifiers are produced in contact space on the body of the listener or through coproduction of classifier handshapes; by prompting, adopting a handshape and then feeling the sign construction (p. 8).
3b. Demonstration	To demonstrate how something is done (for example, describing making a cake), you should use protactile demonstration. Signal the handshape to be adopted and show/create the demonstration together (p. 9).
3c. Mapping (visual pointing to locations in Visual ASL)	Always use protactile mapping when you give directions or describe where something or someone is: use contact space to establish (a) where we are now; (b) how to navigate to the destination (e.g., by tracing a path on the leg) (p. 9–10).
4. Size and Shape Specifiers (SASS) (visual signed languages have constructions known as “Size and Shape Specifiers”)	When describing qualities such as sizes and shapes, each description should be arranged in contact space in relation to the larger thing (p. 11).
5. Exceptions (signaling a move to neutral space before discussing “off-limits” areas for touch)	If the first principle conflicts with cultural norms or is physically unsafe to apply, establish alternative conventions (p. 11).
6. Information source	Include the source of the information when sharing information about events and occurrences (p. 12).
7. Tactile imagery	Protactile* should not be thought of as a way to communicate information to DeafBlind people; rather, it is a means of sharing experiences (p. 14). This requires creating tactile imagery in contact space.
	*Granda and Nuccio use the term PTASL in their original article, as this was an early statement of principles. Following the evolution of Protactile language, the term Protactile is established.

#### 4.1.1 The four articulators of protactile language

A fundamental phonological distinction between ASL and protactile is that in ASL, the two articulators belonging to the monological signer become four in protactile: the hands and arms of both Signer 1 (S1, the speaker) and Signer 2 (S2, the listener) (Edwards and Brentari, 2020). These are denoted as A1–A4 in order of most to least active (see Table 2).

**Table 2:** The four articulators of protactile.

Articulator (hand)	Position and role
A1	Dominant hand of S1is in continuous contact with A4 (non-dominant hand of S2); ASL signs and fingerspelling.
A2	The dominant hand of S2 provides any response from S2 back to S1 (backchanneling), usually on the thigh of S1; it also functions as a proprioceptive object recruited for articulation by S1 to introduce topics and ideas.
A3	The non-dominant hand of S1 creates grammatical structure, often a support for A2.
A4	Stays in contact with A1 to sense movement.

The incorporation of the listener’s body into the process of articulation early on led to several important discoveries and innovations (Edwards and Brentari, 2020):

1. Contact space: the body of S2, and the site of meaning-making and sharing in protactile. Airspace is space in front of, around, or on S1’s body. In ASL, signs are produced in airspace on and in front of the body of the lone signer and are perceived against the backdrop of the signer’s body. In tactilized ASL, the addressee has access to the hands of the signer (S1 has Hand 1 in “speaker” position while S2 follows with non-dominant hand resting on the signing hand of S1 in “listening” position) but not to the visual backdrop against which signs can be distinguished (granda and Nuccio, 2018). In contact space, signs are made clearly perceptible on the backdrop of the listener’s own body.
2. Protactile requires constant contact between S1 and S2 as contact space is the necessary backdrop against which to perceive both successful and unsuccessful communications (granda and Nuccio, 2018) and the space in which emotional states and reactions of interlocutors can be registered, as well as description, demonstration, depiction, back-channeling and direction-giving (Edwards and Brentari, 2020; Lu et al., 2024).
3. Recruitment by S1 of specified areas in contact space evolved from inviting S2 to co-articulate signs (Edwards and Brentari, 2020) to conventionalization of specific grammatical tasks for the four articulators (A1–A4), just as Hand 1 and Hand 2 in ASL have specific tasks.
4. The proprioceptive construction (PC) and the proprioceptive object (PO): The PC is a four-articulator construction (hands and arms of S1 and S2) that has four communicative functions produced by all four articulators in temporal order: initiate, proprioceptive object, prompt-to-continue, and movement-contact type (MC). It uses space to represent spatial relationships, and this space is always contact space.

#### 4.1.2 The proprioceptive object

In protactile, “proprioceptive” refers to the articulatory space (contact space), and the Proprioceptive Object or PO is the articulatory shape formed by using A2, or the dominant hand of the listener/S2 (Edwards and Brentari, 2020, p. 828). The PO has 2 functions: it conveys information about size, shape, and position, and circumscribes and activates a space on the body of S2 (contact space) on which S1 can produce signs (Edwards and Brentari, 2020). A crucial point here is that, while S2 appears to produce “handshapes” (A2 activated and supported by A1 and A3), what is produced in fact is a proprioceptive means for S2 to perceive shapes and their positioning (*Ibid.*). In 4-handed constructions, then, the activated articulator (A2) functions both as a space for articulation and as a “backgrounded, meaningful element” which is represented as a PO. MCs or movement contact types describe the action of the established PO or an additional PO, thus backgrounding the previous PO.

#### 4.1.3 Significance for people w/cdb and their support partners

One of the most important contributions of protactile language is its grammatical resources for epistemological alignment (deixis) and intersubjective engagement as an alternative to mere pragmatic or practical strategies for this engagement (Edwards, 2022).

The grammaticalized use of the proprioceptive channel is of particular significance for linguistic communication with and by People w/cdb given that the bodily-tactile is their strongest channel. “Object” for visual and/or auditory people whose early perceptual history is one of distal access, like the initial developers of protactile language, is a discrete and identifiable aspect of the world. As such, an object makes itself important to us through our interactions with it, for what it does coupled with what we can (and/or must) do with it (what it affords; Gibson, 1979). People w/cdb may be uninterested in any but a curated number of these, making shared activities for meaningful communicative exchange particularly challenging to identify (Peltokorpi et al., 2024).

The object (any object) in the sense of a discrete material, human-made thing is a cultural artifact (extends some aspect of a culture) that only exists in contexts of use, practice, and reference (Malafouris, 2014) to which the Person w/cdb has only fragmentary and usually heavily mediated access. For distal people, including those who have become fluent protactile users, the world is made up of things like these—in many ways, the “world” is these things. In contrast, the world for the Person w/cdb is likely to be one in which surfaces are more salient than the objects they belong to. A toy that makes a vibratory movement is not in itself necessarily of interest or even perceivable to the Person w/cdb as such—as a “toy,” even though the movement it produces is; it requires much consistent exposure to motivating experiences with things to establish even a baseline of continued interest in them and therefore in “thingness” (Miles, 1999). Without this, the object is but a theoretical construct, like the moon—a non-existent irrelevance.

Recruitment of the body of S2 in the construction of POs and PCs concretizes concepts and makes connections between what is being communicated by S1 and the experiential context clearer than is possible in air space in the tactile dyad. Also, as an active participant in these constructions, S2 has access to sign parts (articulation) from within a participatory enactive context against which both meanings and the articulatory process can be perceived simultaneously. In protactile, and to a far greater extent than in sign languages, meaning and articulatory process are one (granda and Nuccio, 2018; Edwards and Brentari, 2020). This four-articulator process differs as well from “co-active signing” (where it is the partner who facilitates/directs the making of a sign by the Person w/cdb; Deuce and Rose, 2019). This is because it is situated as part of a reciprocal communicative process, and not a stepping away from the speaker-listener dynamic, however briefly, to produce an isolated sign (as though the sign were the point rather than the communication).

The re-configuration of articulators and articulation along lines of tactile affordance and tactile intuition (Edwards, 2024) revivified conversational communication that had lost its vitality with the loss of sight and the need to rely on tactilized visual sign language (granda and Nuccio, 2018). With changed embodiment came changed being in the world and a new world to be in; translation of visual experience into tactile descriptions of this experience felt “flat” because the former was no longer relevant to real and situated embodied experience, but a fading reminder of what had been and of the distal chauvinism of a predominantly hearing and seeing culture (Clark, 2017).

### 4.2 Protactile language principles and MET

#### 4.2.1 Thingness and thinging: material and intersubjective engagement in meaning-making

A key point we wish to make here is how the principles of protactile language, as a thoroughly tactile language, might help make use of cultural signs more accessible, and beyond this, richer and thus more meaningful for the Person w/cdb. A crucial aspect of embodiment in this regard is the way the grammar of protactile as described above embraces “thingness” (the manner of being of any “thing”) in terms of a relational process and co-construction rather than as an external, fixed product (an object). This recalls Heidegger’s view of the thing not as an object for contemplation, but as a “thing in use” (Heidegger, 1975; in Malafouris, 2014). Malafouris (2021) defines both mind and thing as a process, such that mind denotes minding and thing, thinging (p. 38). He uses *thinging* to describe how things in this expanded sense both surround us and become part of our minds (brain and body): *“Thinging* articulates the process of thinking and feeling *with*, *through*, rather than simply *about* things” (p. 38; emphasis in original). The proprioceptive object, along with the proprioceptive construction to which it belongs, transforms the act of naming a thing, an object, with a sign or word, and brings this act and the thing named to life through the shared articulatory process achieved by the four articulators.

Protactile language makes “doing things with words” (Austin, 1962) a thoroughly embodied AND grammatical intersubjective project, and in doing this, we suggest, shows us how language and signification (including as thought) are projects of enactive, embodied *being* (process), rather than mere products of *embodiment* (fixed, “finished” state) (Malafouris, 2013, 2008). Protactile theory and practice thus move the question of the embodiment of language to a new point of departure. Malafouris’ perspective of Material Engagement Theory (MET) (Malafouris, 2013, 2008) has much to contribute to understanding the utility of protactile for People w/cdb. MET is a broad theory of culture and evolution that is compatible with the tenets of “4E” theories of embodied cognition (Alessandroni et al., 2024; Gallagher, 2023; Malafouris, 2013). It is this latter perspective we wish to highlight as we reflect on how protactile principles can inform both the challenges and potential of cultural sign language use with and by People w/cdb. MET helps us to see why language in its only reality in embodied use really is languaging in the same sense as thinking (and creating and culturing) is “thinging” or situated material engagement.

Edwards (2022) describes the achievement of intersubjective engagement in protactile as a function of its distinct grammar as cultural tactile language. The achievement of engagement through grammatical and non-grammatical means in language use is a common topic in conversation analysis and cognitive linguistics (Evans et al., 2018). In moving beyond grammar in her discussion of protactile theory and contact space as the “medium of intersubjectivity” for protactile people, Edwards (2024) refers to Heidegger’s concept of being-in-the-world as opposed to having a perspective on the world. She draws connections between this perspective and what Kockelman (2006) calls the “constituents” of the residential whole, which correspond to being in the world, are non-propositional semiotic processes, and as such,

“(…) their objects are neither inferred propositions nor concepts, their signs are material features of the world (…) (Kockelman, 2006, p. 22). *Grasping the affordances of instruments in a particular way* will call forth certain kinds of action (and not others); engaging in the kinds of action those instruments afford will make certain roles available, and taking on particular roles habitually will lead to a mode of existence” (Edwards, 2024, Footnote 3; *emphasis ours*).

What is described here is like what Malafouris (2021, p. 44) calls a “cognitive ecology of skillful material engagement,” including through tool use, or a literal grasping of the affordances of instruments that is more an enmeshment or entanglement of human being and material world (Malafouris, 2016). This engagement is no internal subjective experience or state, but rather a human-world dynamic that creates and sustains culture as a never-finished, always emerging material engagement process. As a member of the Protactile DeafBlind community, for example, one is protactile rather than merely using protactile language, for example, in the segregated way in which we normally view manual tool use (Malafouris, 2021).^2^

MET has three hypotheses (Alessandroni et al., 2024; Malafouris, 2013):

1. the extended mind hypothesis (cognitive processes involve co-constitution relations between brain, body, and world),
2. the hypothesis of enactive signification (the unique semiotic characteristics of material culture generate “processes of signification that follow an enactive logic of sense-making”); and;
3. the hypothesis of material agency (agency is the “product of situated activities where materiality has an active role”) (Alessandroni et al., 2024, pp. 89–90).

In the enactive, radical embodiment perspective of MET (Malafouris, 2013), human development, both cultural and evolutionary, is viewed as an “ongoing dialectic of co-constitution of people and things” (Malafouris, 2016, p. 289). Humans are thus embodied *beings*, not merely embodied, and able to change their development by changing their means of material engagement through things and “assemblages” which “scaffold the ecology of our minds and shape the boundaries of our thinking” (p. 290). The dual processes of embodying and material engagement are preconditions for one another, and embodied cognition involves and emerges from situated dynamic interactions between different types of materials and activities. These are “‘extra-neural’—bodily, artefactual, semiotic,” making embodiment an act of embodying and not a quality or a fixed state (pp. 291–292). Importantly, Malafouris takes issue with the Neo-Darwinist perspective of adaptation by moving beyond mere interaction and fit between actor and world to stress the reciprocally participatory and therefore reciprocally transformative nature of this interaction between human body and environment, hand and tool. It is only when these latter two are put together and in motion that they reveal what they are: “no hand is a hand and no tool is a tool before and outside their actual engagement” in a relation that is “trans-actional co-constitution” (Malafouris, 2021, p. 44).

#### 4.2.2 Protactile language as an assemblage

Protactile language can be said to be an assemblage in the MET sense in that it is a continuously evolving system for situated dynamic interaction between protactile community members, involving full use of proprioceptive-tactual resources according to its rules and conventions for semiotic purposes of meaning-sharing and sense-making.

Viewing A2 as a tool from the perspective described above, we can view it as both a hand and a tool-in-use and a product of this transaction. The grammatical roles of the four articulators [the 2 hands and arms of Signer 1 (A1 and A3) and those of Signer 2 (A2 and A4)] afford two parallel streams of information-sharing and meaning co-construction: the linguistic and the meta-linguistic (Edwards, 2022; Edwards and Brentari, 2021, 2020). In their account of the emerging phonology of protactile language, Edwards and Brentari (2020) point out that the increasingly conventionalized connection between form and meaning was not a single grand achievement, but rather one of increasing stabilization. This ongoing practice of forming protactile transcends the mere adaptive one of translating visual signs to fit the tactile modality, to become a transactional one of enactive signification in engagement with the material, extra-neural world. Malafouris (2016) refers to the stage in the production of a clay vase when what has thus far been formed is stable enough to be recognized as a vase, yet also “plastic, open to change and transformation: an experiential mixture of materiality, affectivity, and creativity” (p. 295). Form-making in this way is transactional and participatory because it is creative engagement where the goal is not one of adjusting the ‘fit’ of one thing to another (as in an adaptation perspective), but rather “one of kinesthetic, semiotic attunement and skilled attentive engagement” (Malafouris, 2021, p. 43). From an adaptation perspective, there is interaction between things and processes, but no participation because these pre-exist their adaptation as fixed or complete entities. In what Malafouris calls creative “thinging,” on the other hand, trans-actional co-constitution produces a much messier “entanglement of mind and matter, organism and environment” that is always in process (Malafouris, 2021, pp. 43–44).

Importantly, what has arisen from the work and practice of the creators of protactile and the evolving practice of the protactile community is more than and different from mere access to language through a different sensory channel (Edwards, 2015). As an emerging language fundamentally distinct from ASL, protactile, as a system of enactive signification in the MET sense, is (along with this) a cultural practice (Edwards, 2022). Protactile provides a good example of how language-in-use is material engagement in this embodied-embodying sense. To paraphrase Malafouris (2014, pp. 142–143), human language, like cognition, is a situated (Dewey, 1989), dynamic interaction and one of the activities afforded by and emerging from the nature of human beings as embodying, materially engaging, and enactive processes. This process connects with the world in endlessly varied ways through the contributions made by the unique properties of each “extra-neural” resource. In protactile, this is demonstrated by the ways aspects of the sense of touch and the qualitative experiences it affords become part of the structure of the language and its conventions of use (Edwards, 2024), and not new ways of accessing the visual world by tactile means. Embodiment as embodying is the process that draws these diverse resources and their properties together in the formation of thought, or mind (Malafouris, 2016, p. 292).

#### 4.2.3 The material sign

Alessandroni et al. (2024, p. 91) describe Malafouris’ concept of the material sign as counter to the traditional code model of language, which emphasizes the separation of sign forms from their meanings as a defining feature of language “proper.” In MET, the material sign can be “touched, carried, worn, possessed, exchanged, transfigured, or destroyed” (Malafouris, 2013, p. 95). This description of the sign as a manipulatable thing produced by and affording types of engagement resonates with language-in-use where the “how of the saying” influences both how speakers are perceived and what it is that listeners hear them saying (Gahrn-Andersen, 2019, p. 177, in Alessandroni et al., 2024, p. 91). Protactile language can be viewed as just such a form of engagement with material signs,

“… that shapes how signifier and signified originate from socio-material practices. In this way, material signs participate in the meaning that is inscribed in them while conveying it” (*Ibid.*).

In this conveying, the acting body, through “patterns and rhythms of coordination” in material engagement and the “kinesthetic experiences, muscular memories and skills attached to it,” does not move in a natural versus a cultural way but instead should be viewed as producing an ongoing and relational achievement of situated material engagement (Malafouris, 2016, p. 298).

The material sign can be viewed as a ‘thing’ in the MET sense. For Malafouris (2014), things are energetic form-matter compounds, and creative “thinging” is a flowing continuity between mind and matter (p. 142). There is tension between thinging and representation: in the latter, things (here, signs) stand-for or re-present something else, whereas in thinging (here, languaging), they “stand-forth” or present (*Ibid.*, p. 143). Malafouris coined the term “thinging” “to “articulate and draw attention to the specific varieties of cognitive life instantiated in “actual occasions” (Whitehead, 1978) of thinking (and feeling) *with, through, and about* material things” (Malafouris, 2014, p. 142). Protactile language users similarly are concerned with actual occasions of tactile experience, exploring and expressing this experience with, through, and about the tactile.

#### 4.2.4 From sender–receiver information processing to intersubjective engagement

MET stands counter to traditional and still largely dominating cognitivist (brain-neural), nativist (innate biological), and internalist (internal psychological states) views of cognition and cultural development (Alessandroni et al., 2024). These view culture as acquired information, knowledge, beliefs, and values, transmitted through social learning, stored in brains (which reside, of course, in individual heads), and expressed in behavior and artifacts (Malafouris, 2016, p. 293). In this view, in cognitive development and activity, “information” is encoded in neural structures that produce mental states that affect behavior, and accordingly, language in the same cognitivist terms is an allied encoded lexical-grammatical framework for producing messages that must be sent by a speaker and subsequently decoded by a receiver. Both are dependent on neural (brain) parts and intra-neural processes that present in the world as behavior and as the twinned products of culture and cultural language, and both are based on mental representations and information processing (*Ibid.*).

The sender–receiver, decoding view of communication is implied throughout the simplified practical descriptions of protactile intended for potential users and/or co-navigators (the protactile alternative to an interpreter or intervenor). This is likely to be because of an interest in producing general and easy-to-understand accounts to facilitate practice for newcomers, and the concern to document the new language in terms of emerging linguistic grammatical conventions and thus as language and not mere “communication” (Edwards and Brentari, 2020). For instance, in a filmed presentation from Protactile Language Interpreting National Education Program (2024), protactile is decidedly not “ethics” but language, the implication being that the former refers to facilitation of communication through caring attention to useful tactile adjustments and translation of visual ASL (Protactile Language Interpreting National Education Program, 2024). However, the ethical implications for communication for People w/cdb and their communication partners, of the protactile demand for observation of contact space in tactile communication, including the principle of reciprocity and the co-presence of these enactments, are considerable.

In protactile, all linguistic communication occurs in contact space, and this undergirds the other six principles. In the requirement to maintain co-presence through adherence to the principle of contact space and the use of “back-channeling” feedback, stance markers (interlocutors’ perspectives toward what is being communicated) are made reciprocally available to all interlocutors. Deixis (that which is being communicated and its source) is clarified and grounded through proprioceptive spatial constructions (Edwards and Brentari, 2021). Intersubjective coordination and engagement are thus achieved through the grammatical use of the hands and other parts of the body in contact space to fulfill the principle of reciprocity through 2-way communication (Edwards, 2022). This literal and enactive (not to be confused with enacting in the pantomime sense) way of doing intersubjectivity is likely to be far more convincing to People w/cdb, in its concrete (materially engaged and engaging) reality, than is the reliance on airspace in tactilized signs using speaking/listening hands. This is not to suggest there is no role for tactualized signs with People w/cdb. They have quite different sensory biographies and thus inhabit a distinctly different experiential world than that of DeafBlind and protactile community members, even compared with the variety and individuality they also have as distinct individuals. Rather, in observing protactile principles (especially contact space and co-presence), the fragmentary deployment of individual visual signs in tactilized form ungrounded in airspace is instead backgrounded, while continuity of co-constructive communicative contact is foregrounded.

## 5 From signal mode to conversation mode in communication

The defining feature of this reciprocity for both communication partners is that it moves us definitively away from monological sender–receiver models of message/information delivery and toward the dialogical: actual conversation. Observance of protactile principles of contact space, reciprocity, and co-presence secures interlocutors’ reciprocal status as equal partners in meaning sharing and construction, and this is only possible in a conversational mode of exchange. Importantly in this regard, its grammatical structures delineate conventions for how the signal or sign-as-code is there to serve this mode of intersubjective engagement and not an end (a sent/delivered signal, code, or message). We have often observed this within the use of AAC, where, for example, the digital voice of a communication aid is met with immediate, enthusiastic acknowledgment by communication partners, while the child’s own “authentic” expressions, when noticed, receive a far more ambiguous response or no response. What is often consistently foregrounded in communication with People w/cdb is the instrument of communication, whether machine or sign, and not communication in the reciprocal engagement sense. Language exists primarily to serve intersubjective engagement, whether of flesh-and-blood, present-in-the-moment interlocutors or through artifacts like a published article (Fusaroli et al., 2014). Protactile principles necessitate that all communication be predominantly in the tactile conversation mode. Even during brief, practical exchanges between co-navigators and protactile clients during prosaic activities like driving to work, there is no place in protactile practice for the use of the signal mode except as a contextualized tool of the conversation. This point brings us back to tool use and MET.

Like Edwards and Brentari (2020, 2021), the work of Gibson (1979, 1966) on environmental affordances informs the work of Malafouris (2013), to which he adds Dewey’s transactional logic of situatedness. In this view, the tool and hand are not separate entities, but mutually co-constitutive through transactional situations of use. All those working in ‘special education’ will be well-versed in the pedagogical context of the situation (students do not eat lunch, they participate in an “eating/meal-time situation,” for example). All situations are also situations of use for the ‘tool’ of tactile languaging. The practitioner’s reality is the complexity of the daily lives of People w/cdb and that of trying to co-navigate it with them. MET describes the primacy of groundedness in the material situation and highlights its transformational, always unfinished (and messy) nature. Mutual engagement in and with the material situation on protactile-inspired terms in communication, activities, teaching, and learning will make mutually transformative, intersubjectively-engaged being together more attainable.

## 6 Examples from practice

We turn now to two examples from cases we have previously presented in connection with prior studies on cdb and embodied cognition (example 1) and training related to the development of tactile signing (example 2), and review these through the lens of protactile principles. Both examples are of filmed episodes from practice that were originally selected for further analysis based on the autonomous actions of each Person w/cdb. Aspects of partner approach in both examples were of significance to these original analyses.

We revisit the examples with the aim of describing the interactions portrayed using protactile terms and concepts. We need to make clear, however, that we are not protactile experts, and no one in these examples is doing protactile language.

### 6.1 Example 1: M

Costain et al. (2019) examined a short film clip of the first author and a child w/cdb during an interaction on a changing table before going swimming. The film shows M, a 12-year-old with restricted mobility, limb strength and movement, congenital blindness, and auditory processing dysfunction. In the clip, partner K uses an approach to “doing and talking about doing” that is suggestive of protactile features.^3^ M is lying on his back on the changing table, being changed into a short-legged body swimsuit with a zipper that K engages him in helping to do up. M has great difficulty with fine motor movement and can only move his limbs by thrusting them forward and/or upward from his position on the table. He can hold a position such as a leg-lift for 2–3 s, make a pincer form with thumb and forefinger, and a light grip form with his hands without power. M readily hangs on to K’s speaking hands. At the end of the 2-min sequence, M is alone on the table as K goes to fetch his bathing chair, and in the few moments before she returns, he appears to return to the zipper session, producing a string of expressions that seem to be re-presentations of several tactile details from their previous activity. The analysis presented focuses on M’s recycling of several signed elements from their interaction in “rhematic” iterations that show him creating altered and/or entirely new expressions that seem connected to these (Costain et al., 2019).

K speaks vocally to M and uses tactile communication, including signs, in a fluid, parallel manner. Throughout their interaction, Hand 1 and Hand 4 (K’s Signer 1 right hand and M’s Signer 2 left hand) remain in almost constant contact. She maintains contact space with Hand 1 and Hand 4 tracing over M’s head and chest to foreshadow coming actions (such as taking off T-shirt and undershirt, doing up the zipper), showing point-to-point trajectory (start–stop for the zipper) and signing directly on his head (SHOWER) and upper body (SUIT (top), NECK, SWIM, ZIPPER) as well as the back of his hand (WATER).

K repeatedly places M in a “doer” position by positioning his Hand 2 right hand with her Hand 3 left hand to facilitate his participation in zipping up his suit. She uses repetition of vocal utterances and signs, prosody, beats (vocal and tactile), haptic signals (direction of action), and, where possible, exaggeration of sign or gestural movements to heighten their salience and to emphasize touch and movement qualities of what she is talking about. For example, she signs FETCH (Hand 1 with Hand 4) while informing vocally that she is going to fetch his chair “over there” in ways that emphasize the movement iconicity of the sign for FETCH. She makes the sign in the direction of the chair (Hand 1 and Hand 4), followed by two beats while pointing hand-under-hand (Hand 1 and Hand 4) to punctuate (“over/there”). She uses walking fingers (Hand 1) on his right forearm (Hand 2) to show how she is going to GO in that direction and come BACK again.

During the doing-up of the zipper, the index finger and thumb of M’s Hand 2 hand are held on the tab of the zipper head by K’s Hand 3 as Hand 1 and Hand 4 draw it up from waist to neckline. About halfway, the zipper gets stuck, and K adjusts it before Hand 1 and Hand 4 resume zipping. At the top, the index finger of Hand 1, with that of Hand 4, pushes the zipper head into a little open-sided pocket provided for it. There is a “co-signing” approach taken to certain signs, like HELP, during which Hand 2 is half of the sign form (supported by Hand 3) and Hand 1 is the other half.

Throughout the interaction, there is attention to engaging M in the action while informing him about it, and K pauses when M uses vocalization to register his perspective before seeking to re-engage (“impatient” sounds; “raspberry” sounds). K is aware they are late and need to hurry to the pool, so there are competing projects to manage. In engaging M’s attention and facilitating his participation, tactile access is afforded to tactile detail enactively, through a conflation of signs and/or hyper-salient action-gestures with the task itself, so that talking-about flows into doing-together, and the doing involves discovering both phonological elements of signs and their production and tactile details of the materials being handled. Aspects of these can be seen to reappear in M’s solitary run-through before K returns with his chair.

Protactile principles that resonate here include contact space and co-presence, aspects of proprioceptive construction, roles of the 4 articulators (the four hands), tactile imagery, and point-to-point mapping. Reciprocity is shown in pausing for feedback before resuming signing, vocal speaking, and doing (K), and through alternate vocalization and silent attentiveness, as well as willingness to continue being Signer 2 (Hand 2 and Hand 4) (M).

**Figure 1:**
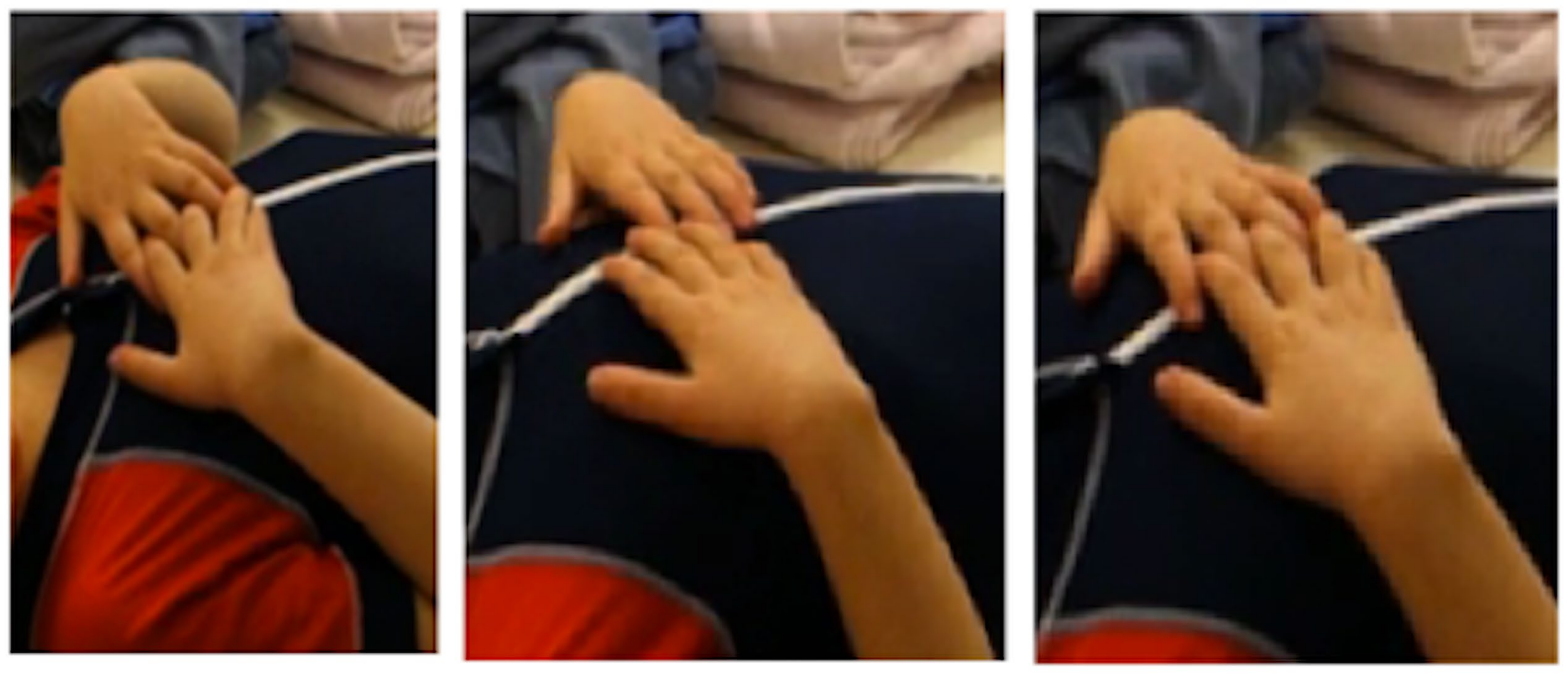
‘Partially closed zipper—open neck suit’ manner on place and ‘closed zipper teeth’ manner on place. The images in Figures 1 – 6 appear in the original article (Costain et al., 2019). We thank the publishers for permission to reproduce them here.

**Figure 2:**
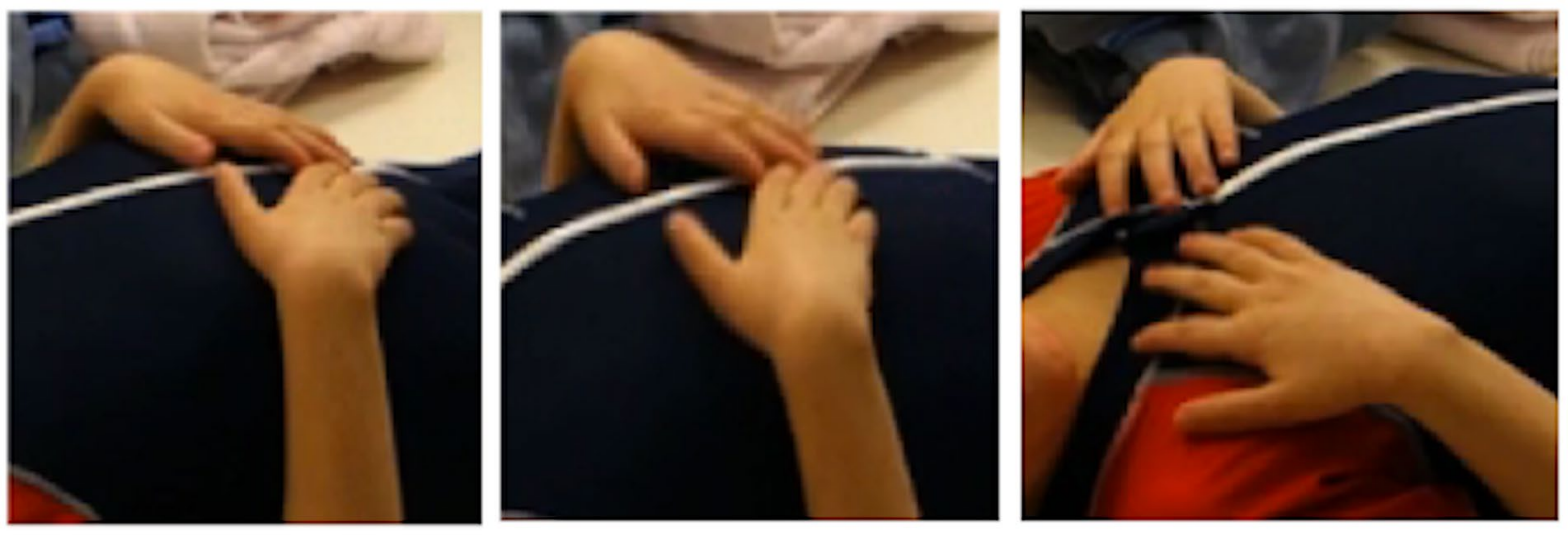
‘Zipping all the way up’ manner.

**Figure 3:**
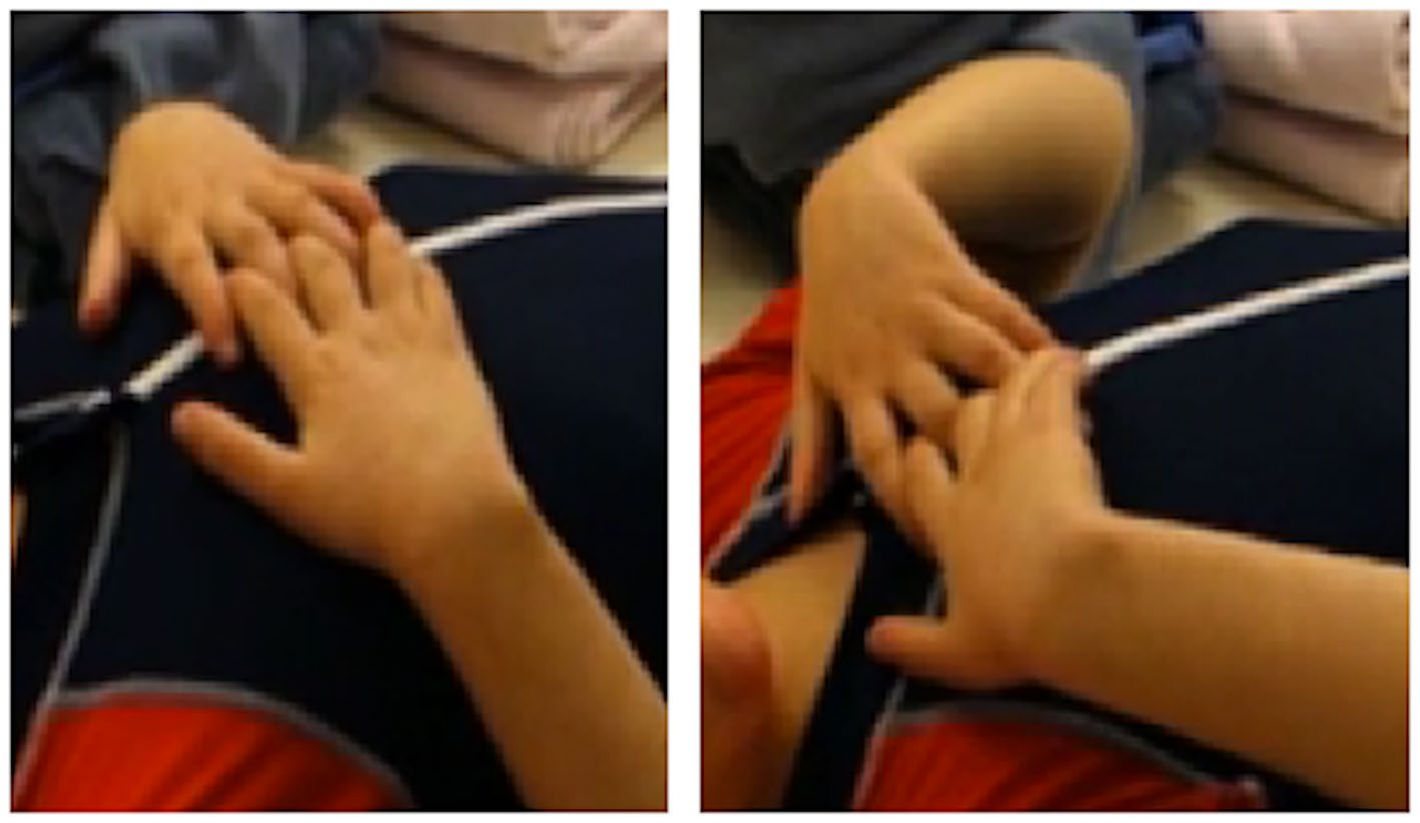
‘Closed teeth’ with ‘zipper’ manners on place = ‘fastened zipper’ manner on place.

**Figure 4:**
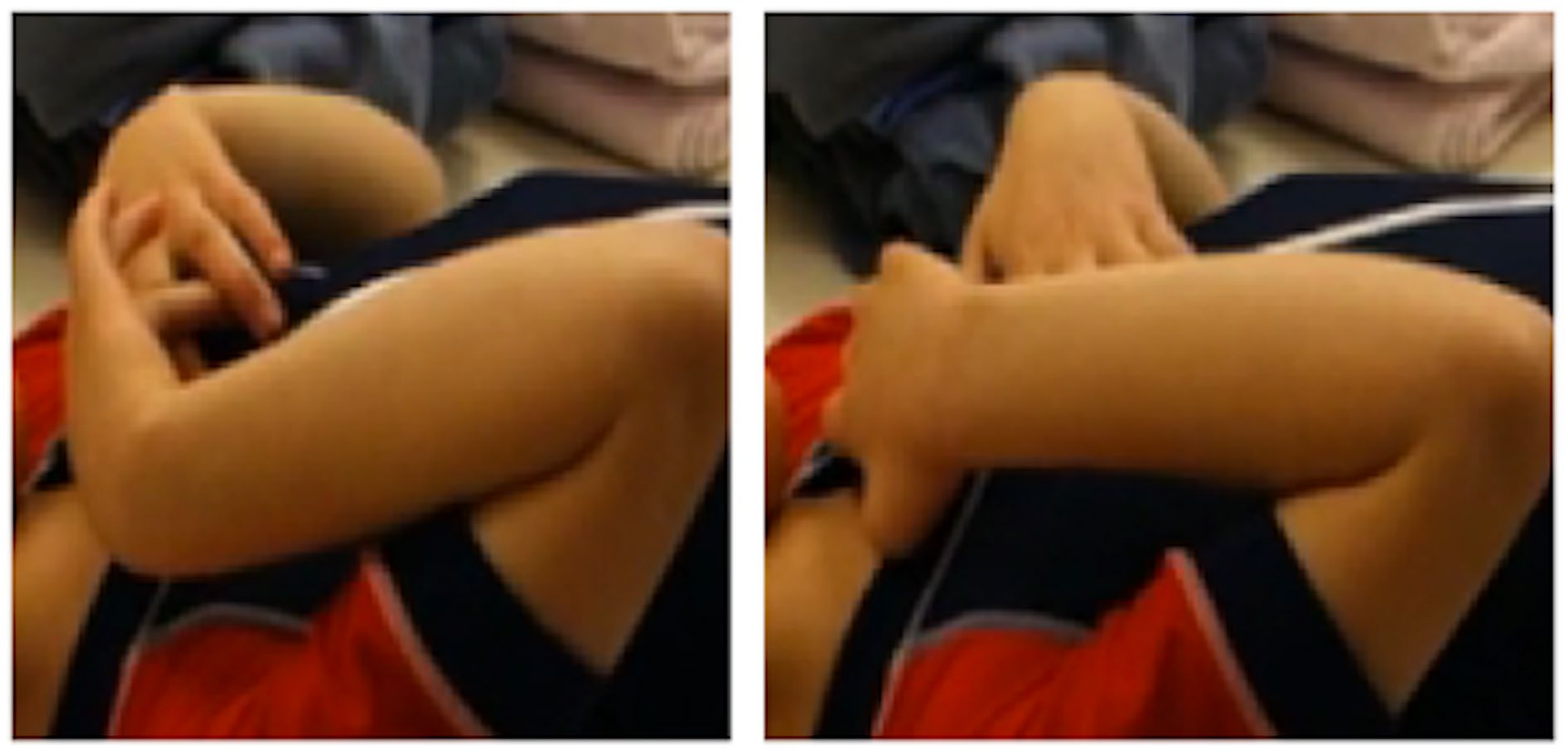
‘Goal’- ‘zipper tab’ manner on place + ‘pocket’ place and manner.

**Figure 5:**
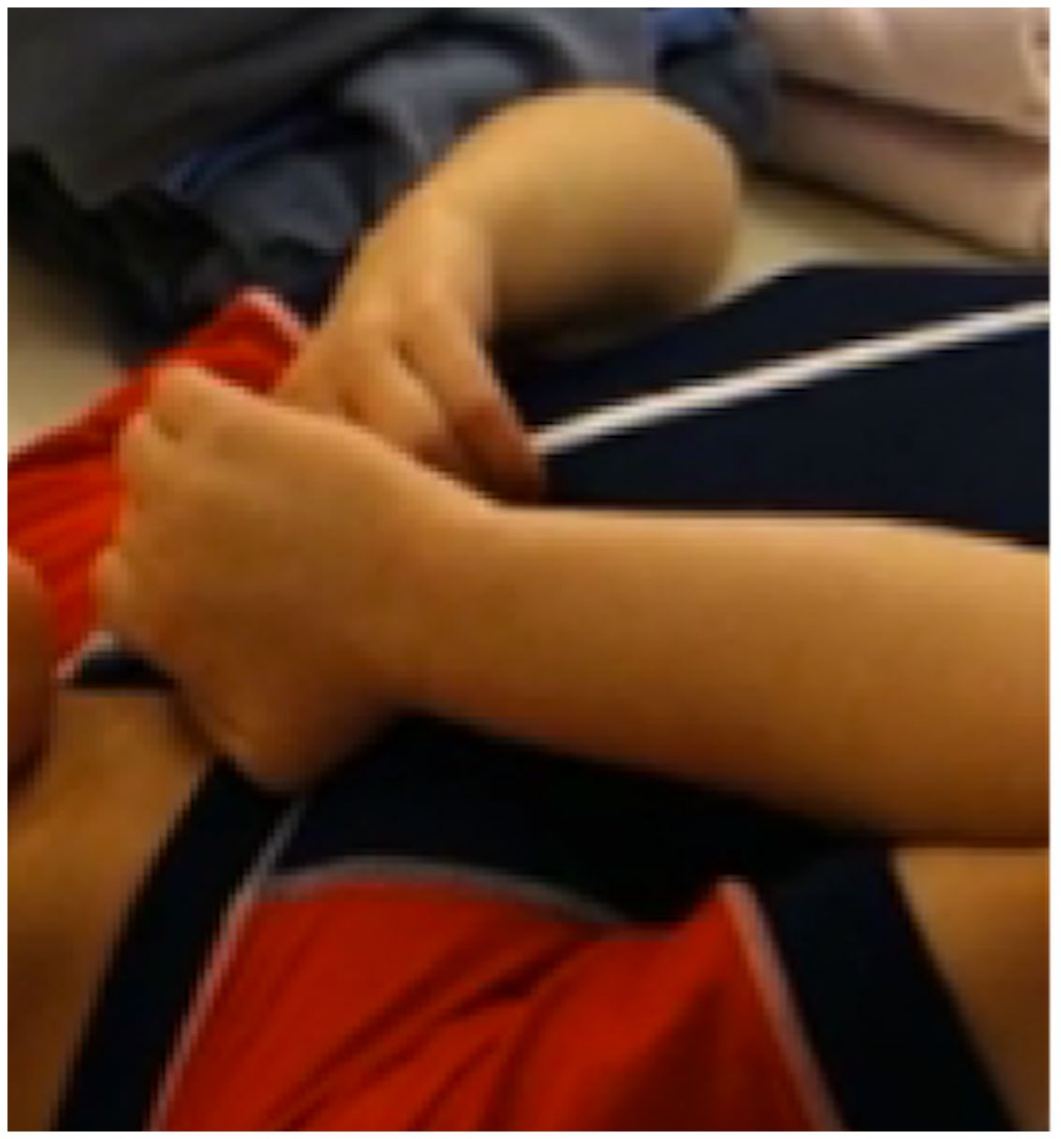
‘Zipper’ manner with ‘in-pocket’ manner.

**Figure 6:**
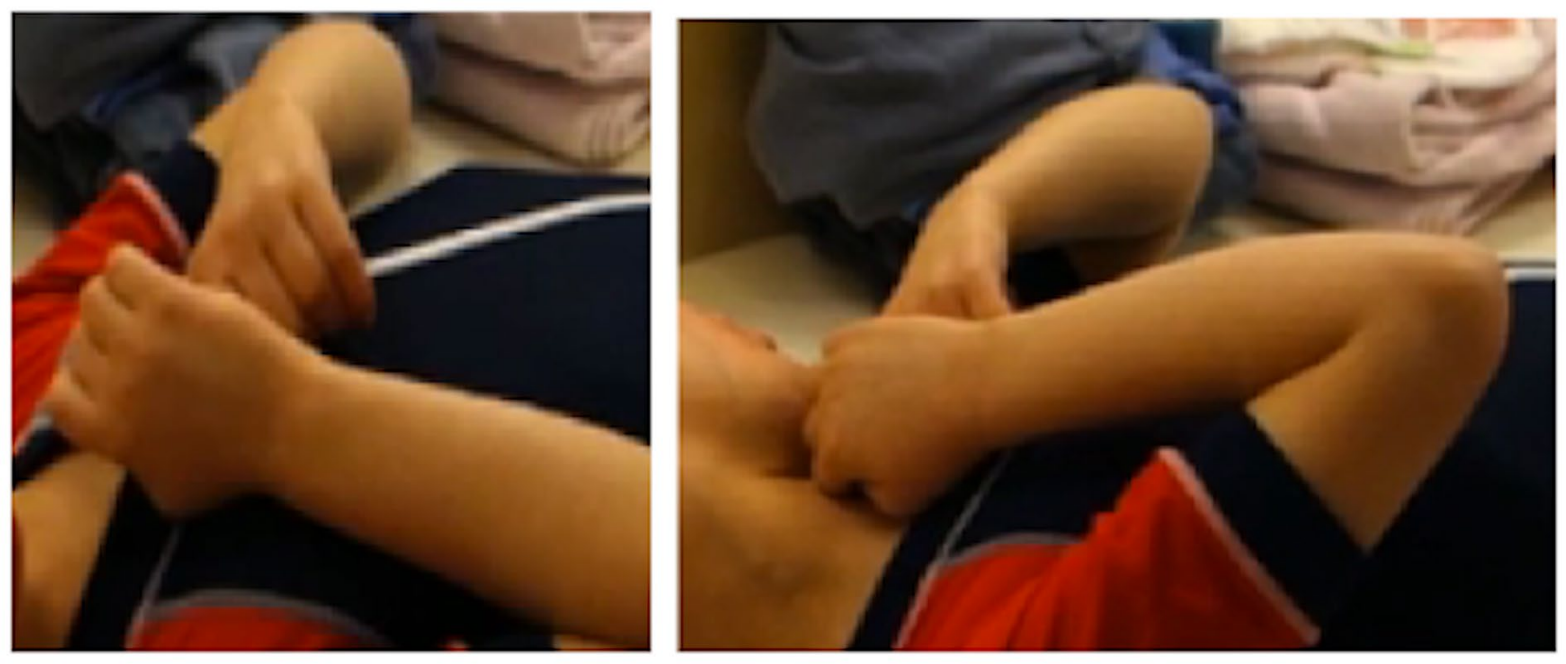
‘Goal’/‘fastening’ place and manner, and fastened up to here (chin) place and manner.

M uses several novel expressions that seem to be connected to both the action in which he took part and the tactile details of the zipper teeth and head, as well as the cloth pocket into which this was tucked once drawn up. M’s revisiting of these details has an enactive quality, but his actions are not a mere re-enactment of doing up the zipper. The analysis of the 18-s sequence shows M’s repeated use of a source-path-goal frame that K has employed with each phase to show M the path from start to finishing point before proceeding with the task and asking him to participate by placing him in the “doer” position. Within each repetition M makes on his own of this start-finish frame, he includes an array of expressions with sign qualities of handshape, movement, and location that seem related to the tactile details of the zipper and zipping. For a child with cdb, restricted movement, and limited exposure to cultural signs, this is an impressive achievement (see Figures 1–6).

Had K merely used the SL sign for ZIPPER in tactilized form, none of this “zipping–zipper manner” would have been perceptible for M, and zipping would just have happened while his attention was elsewhere, like so many other events in his life.

### 6.2 Example 2: F and the emu

The second example comes from recent practice by the second author, who was supporting a young Person w/cdb, F, born with a profound hearing level and reduced vision, and remaining a promising symbolic communicator. F attended an alternative-school setting on a farmstead.

F had become a vivacious communicator, using a combination of cultural, authentic, and mimetic signs to express himself. The tactile-kinetic sense is a core channel of information for him, with tactilized national SL considered his primary mode of communicating. His partners had a range of sign language competence, and F code-switched according to the level of each one. He had exchanges with partners from an advanced level of competence in the cultural sign language (interpreter level), through to partners using key word sign or authentic/home signs.

At the time of this interaction, he was primarily supported by beginner-intermediate skilled signers. The regulation of his sensory system impacted his ability to remain present and engage with others. He demonstrated a keen sense of humor, often challenging the concept of what is ‘naughty’ by testing out what he could do in a social setting. He often jokingly signed DINOSAUR in connection to unplanned events to deny his involvement in them (e.g., ‘I did not do that; the dinosaur did it’).

The events in this interaction were documented during a consulting visit. A short video of an interaction between F and his support worker was analyzed by the second author. This interaction uses the speaking/listening hand positions of tactilized sign language.^4^

#### 6.2.1 Video analysis of the interaction between F and his support worker

##### 6.2.1.1 The event

**Figure 7:**
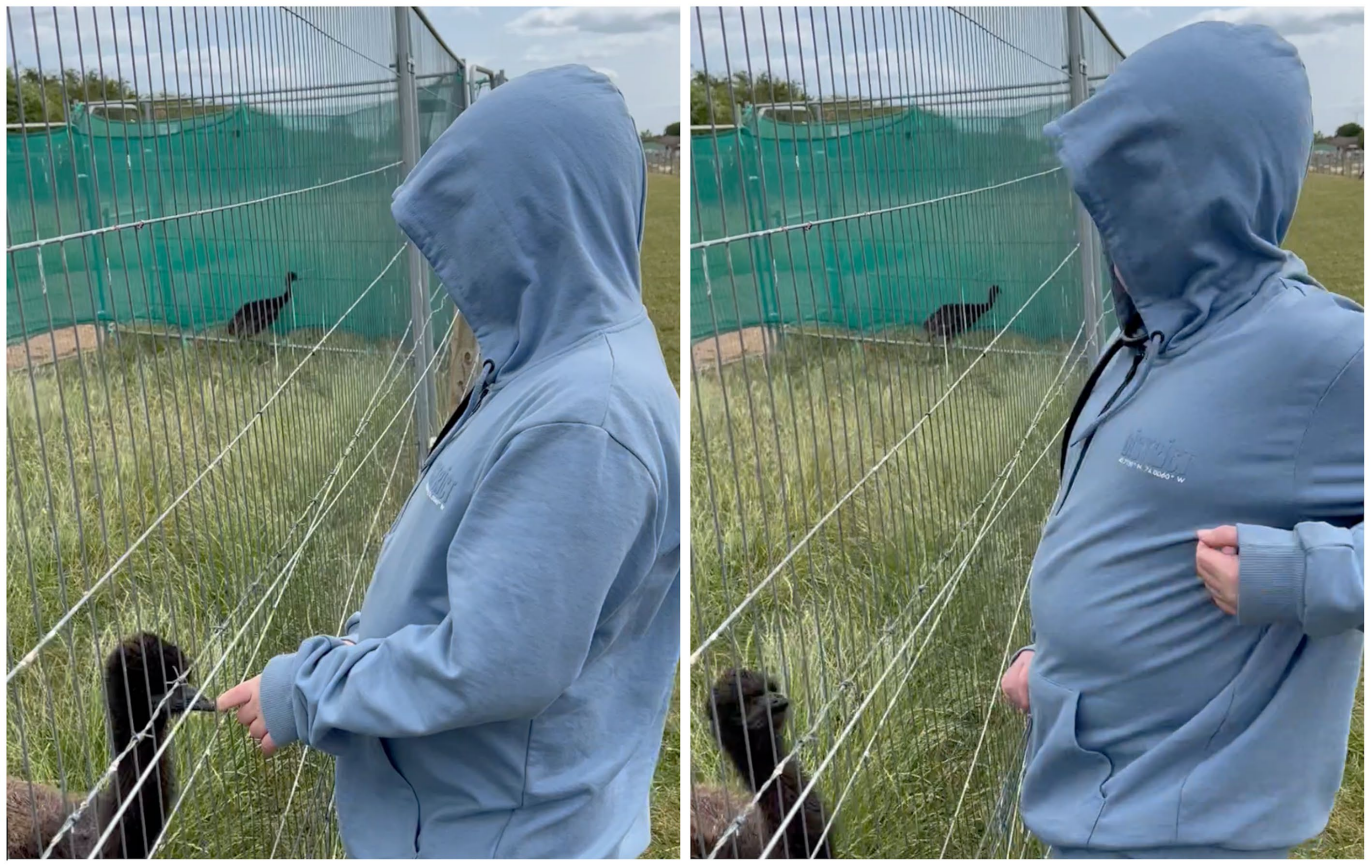
The event. F wears a stethoscope in the pictures. F often finds objects that he attaches to and carries with him during the day. The stethoscope was his object on the day of the interaction.

**Figure 8:**
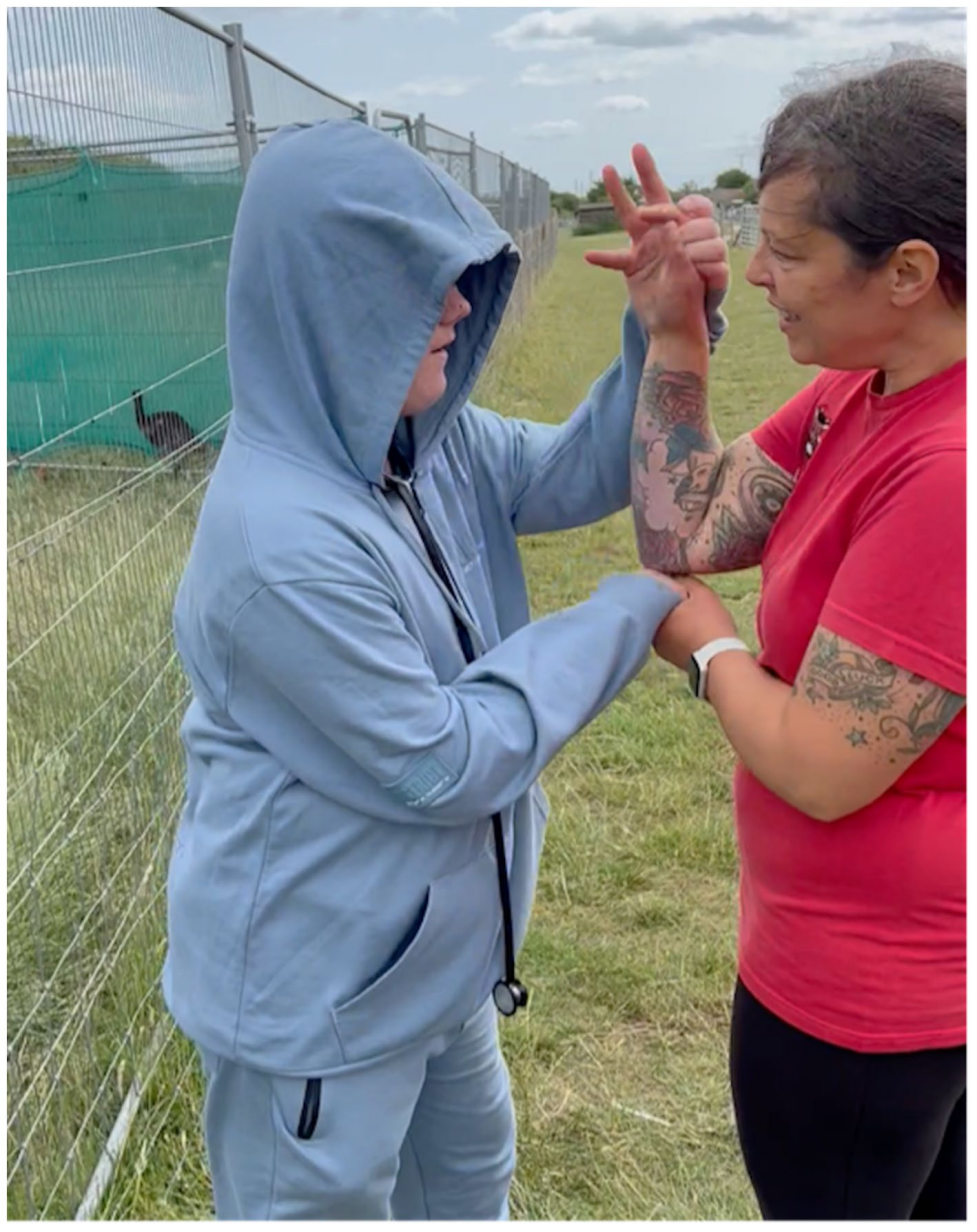
The account.

**Figure 9:**
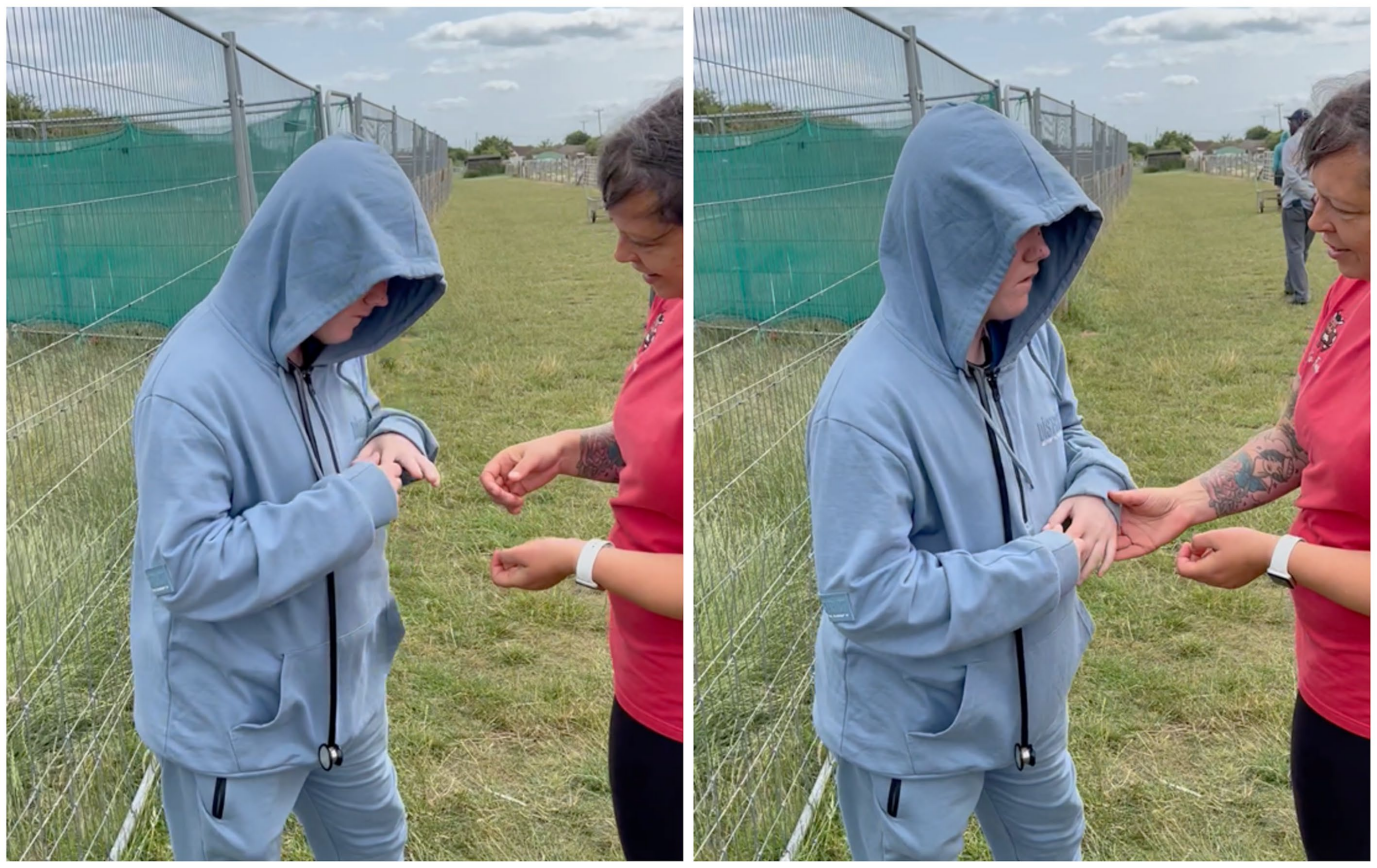
Reflection and back-channeling.

**Figure 10:**
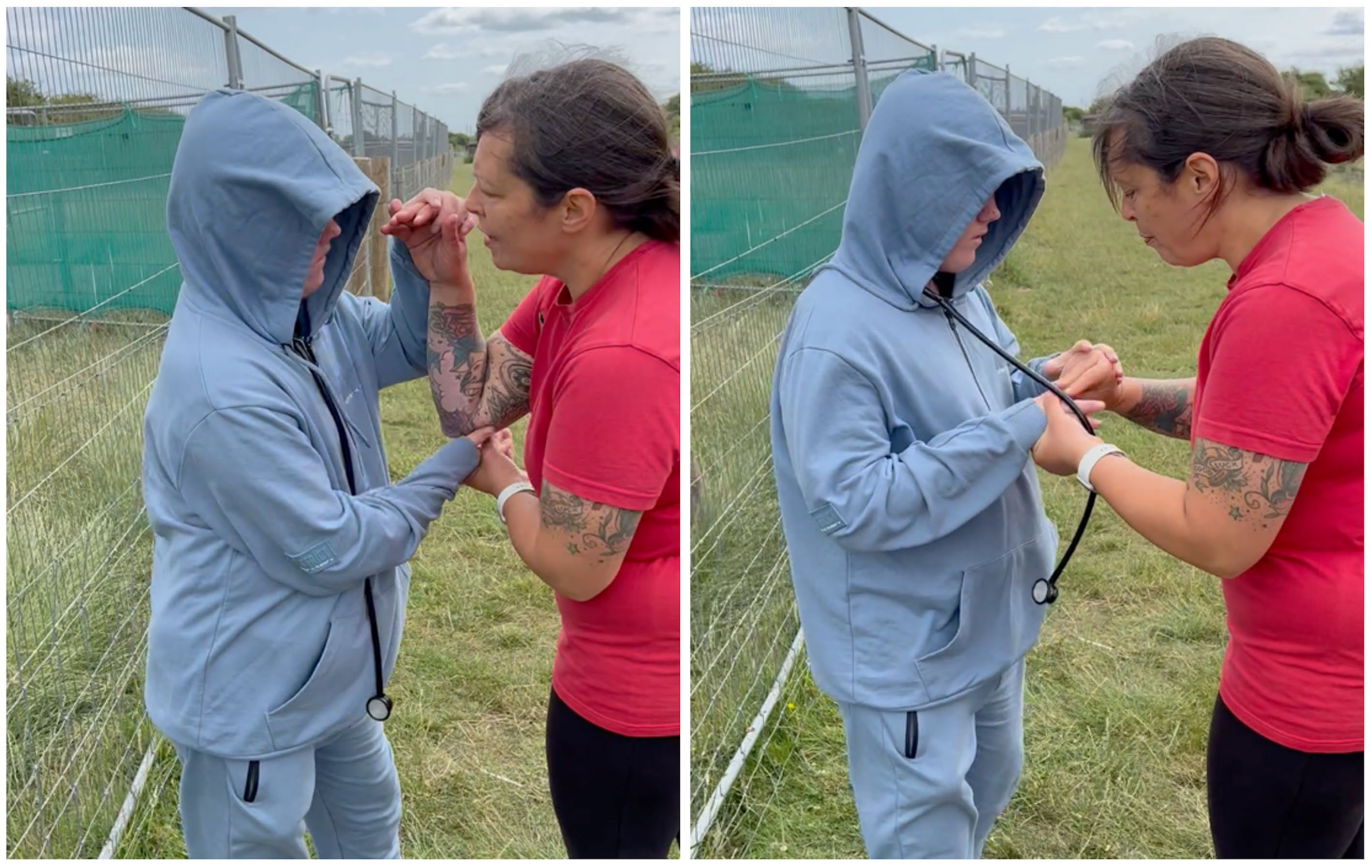
Review of the event.

**Figure 11:**
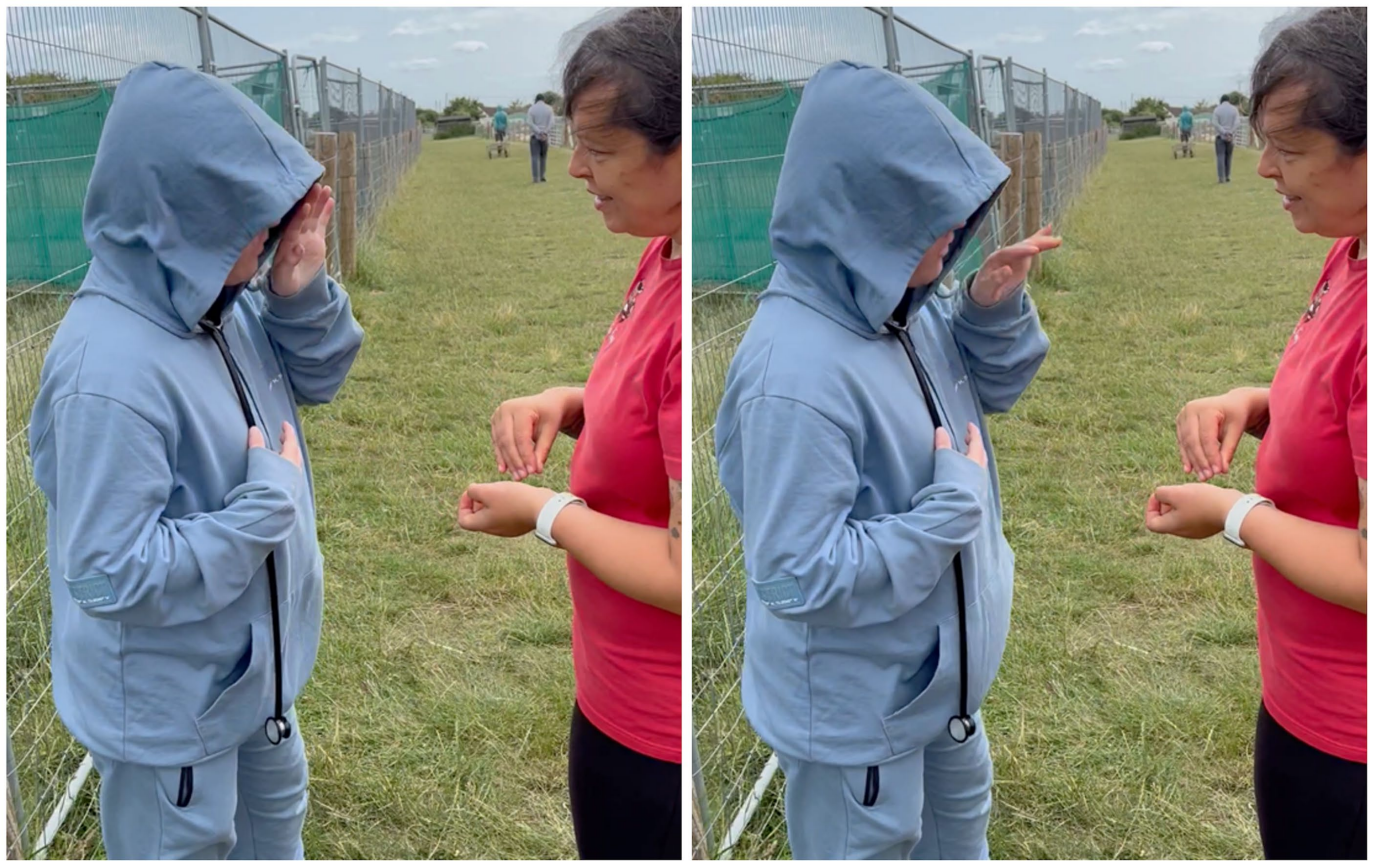
Unrecognized expression.

F is at the emu pen with his partner behind him out of tactile range, and Second Author filming them (presence of both is known to F). F rests both his hands on the mesh fence, tactually orienting to and referencing the fence. He hooks the index finger of his left hand around a vertical wire, positioning his hand in a way that suggests familiarity with what is about to happen, perhaps based on previous experiences of visiting the emu pen. An emu approaches and quickly nips the finger. Immediately, F pulls his hand away, closing his fingers into a fist and pressing it against his body (see Figures 7–11).

##### 6.2.1.2 The reflection

As he relaxes his hand (LH), he rubs thumb and index finger together (a first expression). From a visual perspective, this withdrawal might appear as a response to pain. However, this nip-withdraw-relax-rub sequence also describes a fully tactile experience and illustrates the creation of a bodily-emotional trace (BET) (Daelman et al., 2004).

##### 6.2.1.3 Initiation of conversation

F turns using his dominant RH, waving in space, and stepping around, as if seeking his partner. From a protactile perspective, he could be perceived as seeking co-presence. His partner receives this seeking movement by placing her right hand on his body/stomach. F stops walking at this point, and his partner assumes ‘speaking hand’ positions, placing both her RH and LH under his LH and RH (noting that this hand configuration differs from the contact-space connection proposed in protactile linguistics and instead reflects a tactile signing dyad). This does, however, provide the opportunity for signs to be made partially in mutual contact and allows co-presence to be re-established.

##### 6.2.1.4 Supporting the conversation

F’s partner initiates conversation, both vocally and with tactilized sign, stating WHAT and EMU. F receives these signs with his LH, feeling his partner’s RH sign EMU. Her RH creates a handshape and becomes the emu. At the same time, F’s RH is resting under the elbow of her RH arm. This ‘anchoring’ places the emu in a type of contact space, F feels there is a base to the emu entity, and (perhaps) that her arm has become the emu in this description. His partner brings her hands down to a neutral space (positioning her hands just in front of her body). This alone signals that she is terminating her turn. F removes both of his hands from the listener position/co-presence with his partner.

##### 6.2.1.5 A second reflection

F places the index finger of his LH against his lips and presses, then puts the finger between his lips, and nips it with them (a second expression; a possible ‘thinking gesture’, my finger was bitten; placing himself as the agent/emu, doing the nipping). He then removes his LH and brings his L index finger into contact with his R thumb and index finger in a pincer grip and uses them to grasp his L index finger at the same place he was nipped by the emu. His partner continues to watch him.

Here, F is showing what happened, and this expression seems to have high meaning-making potential directly related to his experience of being nipped by the emu (which is also a shared experience with his partner). His RH has tentatively become the Emu, repeating (recycling) the nipping. His partner establishes co-presence by offering an open RH under his LH. His RH continues to repeat the nipping/pincer action, as his partner taps several times under his hand, signaling on his body that she knows what he is showing, (back-channelling).

##### 6.2.1.6 Revisiting the narrative

The partner initiates and scaffolds further conversation by taking his LH with her LH, prompting him to assume the listener position. As she brings her RH under his, she contacts his RH with her LH, this time prompting his hand (pulling his hand, coactively) to touch her R elbow (the base of the EMU sign). Simultaneously, her RH becomes the emu, and F rests his LH on her RH. F then steps forward and pulls his hands away. His partner follows, making continuing contact with his RH using both her hands. This appears to be slightly directive, using pressure or resistance perhaps to get F to stop walking. She then readjusts her position, directing both of his hands to a listening position.

The partner then signs EMU, adopting sign positioning as previously described, then takes a different approach when her RH becomes the Emu. She uses the emu handshape and brings the beak (fingers of her RH) down to the fingers of F’s RH and begins to nip (‘Your fingers were nipped by the emu’). In protactile terms, this suggests a tactile perspective and cocreation of tactile imagery. Both interlocutors are using their hands to actively co-construct the image of the emu nipping F’s hand. In talking about the event, F’s hand being nipped (RH) is not the hand that was nipped in the actual event (LH); shared meaning seems to be achieved. F lets his partner know that he knows what she knows (that he was nipped by the emu), when, following this co-constructed image, he withdraws his hands but then makes contact again, leading with his RH. His partner receives this initiative and repeats nipping of fingers with her emu hand (RH). F then pulls his RH away and holds it close to his chest, as he had held his LH during the event itself. His partner releases his hand, and F then offers a new initiative. He makes a hand gesture using his LH, a ‘relaxed’ handshape positioned next to his face, oriented with palm toward his partner. This hand then moves in a slightly upward/forward-downward arc. This gesture is not acknowledged in the interaction with his partner.

## 7 Discussion

We will reflect on these examples from non-protactile practice to highlight the potential that adoption of protactile principles can have in enriching meaning sharing with People w/cdb, and how these principles can inform the perspectives support partners have of their own languaging activities. We conclude with a pragmatic suggestion for future directions.

### 7.1 Recycling language parts and tactile imagery

M’s expressions were analyzed as recycling with différance (Anward, 2004), in which each round of recycling of a previous conversational turn or element introduces or “sets into play” a difference in Derrida’s (1981) sense, “as it unfolds, or is constructed in time” (p. 10, in Anward, 2004, p. 26). Anward remarks that in recycling with différance, speakers are doing syntax (fitting turns with syntactic structures) as well as doing other aspects of grammar in the pragmatic world of languaging, such as,

“(…) deriving a new word from an old word, creating a compound involving old lexical material, promoting old lexical material from part of a compound to an independent word, extending a word or a larger unit to a new function, and finding a semantically related expression for an expression used so far.” (p. 33).

Similarly, F’s partner responds to him by recycling his conversational turns and expanding her repertoire of movement and action in her signing and tactile communication accordingly to deepen shared meaning.

This is strongly reminiscent of accounts of early development of protactile language, starting with salvaging from the “junkyard of ASL parts,” moving on to new language-building to reflect increased experiential habitation of a tactile world by interlocutors (Edwards, 2015; granda and Nuccio, 2018). Protactile moved away from salvaging parts to actively recycling what was serviceable within the new demand for tactile salience and grounding. Recycling as a feature of conversation involves repetition (in terms of re-use of prior turns or elements) but can be generative as well as reductive in the creation of a range of expressions from “strongly reduced (hypo-) to over-articulated (hyper-) versions” (Anward, 2004, p. 34; Linell, 1979). This poetic mode of recycling one another’s contributions supports deixis and engagement in a pragmatic, intersubjective flow of inter-action, and as such, makes the conversational project one of elaborated perception (Costain et al., 2019).

In no other form of languaging, perhaps, is this so thoroughly embodied as in protactile languaging, which has as its medium the joint linguistic-communicative task or project of embodying shared meaning in the “thing-ing” sense of material engagement described in MET. Recycling is not a fixed entity to be wielded (a tool for adapting ASL for tactile expression) but a continuous playing out of engagement with what has been articulated or is being articulated in a (forever unfinished) thoroughly tactile conversation that generates novelty while retaining aspects of previous turns and their elements. Here we refer to both the continuing development of protactile language and its practice, because to separate these two throws us immediately back into a dichotomy of mind and matter that becomes irrelevant from the perspectives of both protactile theory and MET.

### 7.2 The sign as a tool

Through the scaffolding K provides by placing M’s hands in “doer” position, he may have discovered new ways to use them, aspects of which are revisited when he produces expressions reflecting the action they have just taken part in. By being “used” in the task of doing up the zipper, M’s fingers are “made aware” of details previously imperceptible to him. In being used by H1 and H3, H2 and H4 are placed in a “doing” position and are thus objectified. M’s autonomous, unprompted yet detailed run-through afterward is not a re-enactment of the task of zippering but rather a narrative series of close-ups of the tactual details experienced by his fingertips held in place and manipulated during the zippering. They can be seen as articulating tactile imagery. At the same time, through the lens of protactile languaging, K’s actions with her own and M’s hands can be seen to partially reflect implementation of the grammatical roles assigned to the 4 articulators and the principles of contact space, tactile demonstration in point-to-point mapping, and proprioceptive construction. It seems clear that a deliberate, foregrounded, and consistent adherence to protactile principles (including, to an extent, its grammar) in communication with M might also support an enculturation of his hands for further linguistic expression, as well as highlight for partners the linguistic qualities of his expressions. A protactile approach, if consistent enough, could provide a more solid shared reference base for M and his partners and improve the ‘readability’ of each for the other.

Malafouris (2014) points out that hands and tools make each other and refers to the “enactive participatory logic of enskilment” that is crucial to understanding this. He references Heidegger’s essay, “The thing” in the phenomenal power of things-in-use to “gather” their physical constituents, space, and time (Heidegger, 1975, p. 166 in Malafouris, 2014, p. 141). Protactile practices can be thought of as a cognitive ecology of skillful material engagement in the MET sense. Protactile interlocutors have evolved a way of making sense linguistically that remains true to and is fully connected to the tactile world they now inhabit. The articulators in contact space are the tools of this sense-making, and the emerging grammar of protactile language is also “ready-to-hand” as a new productive enmeshment, or entanglement in the further evolution of the language. For our purposes, however, it is sufficient to note how consistent use of protactile principles in tactile communication necessarily foregrounds tactile imagery. This would support further enculturation of the hands for articulatory expression and, more importantly, make clear the link between signing and exploring in gathering information and sharing experiences.

### 7.3 Moving from “readability” to linguistic-conversational salience in contact space

Tactilized signing produces an impression of sign-parts for the listener that will not match the visual sign, or the speaker’s tactile production; the sign “as read” in air space will lack clarity and will have a different form. F appeared to use a version of ‘EMU’ that matched the way the sign felt to him when his partner signed it. In listener position, his LH rested on top of his partner’s RH, so when he adopted speaker role (in air space, since his partner did not place her hands on his to listen), his expressive sign remained closely related in form to his experience of the articulatory structure of her signing it to him: location (positioned the start point in relation to his face similarly to his partner’s start point), movement path (handshape moved upward, forward, then downward), and adopted the handshape that he perceived through his passive listening position (an open, relaxed hand). Hence, when he came to make this expression, his articulation was that of “sign as perceived” (Forsgren et al., 2018). This expression is overlooked in the interactional context, suggesting that the articulatory specificity of the sign-as-read and then produced in air space reduces readability for his partner. Keeping the interaction in contact space or at least following his signing by placing both her hands in listener position would have made her perception of the sign clearer and increased readability.

The readability perspective is one of decoding a fixed sign, whereas salience is one of following what is being “said” about the tactile world from a tactile perspective and along tactile lines. Protactile gives the phenomenon of signs-as-read priority and thus accords authentic, or self-created expressions, genuine linguistic status lexically and/or grammatically. Protactile languaging stresses this innovation and the use of the tactile environment in the moment, as F does here. When this can be seen as part of formal linguistic meaning sharing, expressiveness can more easily be seen by partners, and responses to it are enriched. The evocative-emblematic mode of how People w/cdb use tactilized visual signs suggests the use of a tiny repertoire to achieve many communicative goals. This contrasts with the specificity in salience terms of F’s expressions, and in the multiple intersubjective projects served by it that can be seen in his self-initiated conversation about his emu encounter. A protactile perspective highlights salience in communication (and communication itself) as a linguistic-conversational, not just an interactional, project.

F and his partner demonstrate the drawbacks of the traditional signing dyad and perception of signs in air space, but aspects of proprioceptive co-construction in the same exchange using contact space are also demonstrated. Turning the air vs. contact space ratio around in proprioceptive co-constructive signing is one clear way of enacting protactile principles.

## 8 Limitations and future directions

One limitation of our article is that it introduces many complex concepts and two novel philosophical perspectives on embodied meaning-making that all warrant more thorough treatment than we can give them here. Although it has been necessary to cover a great deal of background in this way, we are aware that this is only the starting point. Also, the examples we have described are drawn from previous studies and not part of a study of protactile practice, which would have given our suggestions regarding the usefulness of the perspective with People w/cdb greater weight. An important direction for further investigation is case studies in which protactile principles are applied from the outset and results evaluated to assess whether protactile brings as much to the expansion of communication and languaging with this group as we have suggested, and in what specific ways.

## 9 Conclusion

Protactile unlocks the proprioceptive channel (the articulators in contact space) and generates more material on which the linguistic system can operate (Edwards, 2024) or with which it can engage. The imperative of maintaining contact space consistently produces a set of consequences for the conversation partners that are further articulated in each of the subsequent protactile principles. Imagery, reference, and feedback must be tactile (felt) by both interlocutors, as communication cannot achieve the level of real conversation without this reciprocity. The adherence to contact space and the consequences of keeping within it, including the production of proprioceptive constructions, can expand the resources for languaging between People w/cdb and their partners considerably. The requirement of reciprocity in a truly tactile mode guided by protactile principles can ensure that communication remains dialogically framed. When such requirements are more consistently met, sign constructions, whether cultural or authentic, will always be co-constructions, highlighting co-constructive signing as the medium for dialogical meaning-making. Traditional coactive and on-body signing approaches in practice to support People w/cdb are vulnerable to becoming directive and unidirectional, resulting in communication that becomes mere information-giving. The modifications that result from the observance of protactile principles should enrich the knowledge bases of both partners through knowledge creation, where mutual access, reciprocity, and engagement with the tactile world create a rich(er) cognitive, communicative, and experiential environment.

## Data Availability

The original contributions presented in the study are included in the article/supplementary material, further inquiries can be directed to the corresponding author.

## Footnotes

1 AAC is the field of practice addressing different ways to communicate in the absence of spoken language. It includes ‘unaided communication’, such as body language, gesture and manual (sign) approaches, and “aided communication,” such as picture or object symbol systems and electronic/powered communication systems. Narrow interpretation of AAC may only recognise powered systems as AAC. Cultural Sign Languages sit outside of the scope of AAC since they are separate, recognised languages. However, manual signing systems are included in the scope of AAC (Comino et al., 2025).

2 Indeed, protactile language is only the fourth element in the Protactile movement (the first three being history, philosophy and attitude; Protactile Language Interpreting National Education Program, 2024).

3 K is not doing protactile languaging, however we use Hand 1–4 to indicate similar roles for the articulators.

4 Where for two right-hand dominant signers the following hand positions are assumed: Signer 1 dominant R hand (underneath) connects to Signer 2 non-dominant L hand (on top), Signer 1 non-dominant L hand (underneath) connects to Signer 2 dominant R hand (on top). Speaker-listener roles are determined by hand position with a change of position denoting turn shifts between speaker-listener in the dyad (Deuce and Rose, 2019; Willoughby et al., 2018).
